# Knowledge gaps about the diagnosis and treatment of hypothyroidism: an international patient survey

**DOI:** 10.3389/fendo.2025.1663497

**Published:** 2025-08-29

**Authors:** Petros Perros, Alan Poots, Endre Vezekenyi Nagy, Enrico Papini, Harriet Hay, Juan Abad-Madroñero, Amy Tallett, Petrus Lakwijk, Laszlo Hegedüs

**Affiliations:** ^1^ Translational and Clinical Research Institute, Newcastle University, Newcastle upon Tyne, United Kingdom; ^2^ Picker Institute Europe, Oxford, Oxfordshire, United Kingdom; ^3^ Division of Endocrinology, Department of Medicine, Faculty of Medicine, University of Debrecen, Debrecen, Hungary; ^4^ Department of Endocrinology and Metabolism, Regina Apostolorum Hospital- Lifenet Health Group, Rome, Italy; ^5^ Thyroid Federation International, Hoofddorp, Netherlands; ^6^ Department of Endocrinology, Odense University Hospital, Odense, Denmark

**Keywords:** hypothyroidism, knowledge, misconception, misinformation, questionnaire, survey

## Abstract

**Introduction:**

Over-diagnosis and over-treatment of hypothyroidism is a growing concern. The role of patient knowledge has not been previously investigated. The aim was to explore patient knowledge in relation to diagnosis and treatment of hypothyroidism.

**Methods:**

Cross-sectional, international online survey. Participants were people with treated hypothyroidism amounting to 3421 valid respondents from 68 countries. A questionnaire was used, which included knowledge statements about hypothyroidism relating to recommendations by international guidelines. The principal knowledge statement was “A patient with a normal thyroid blood test does not need to be treated with thyroid hormones (even if they have positive thyroid antibodies and symptoms)”, and participants were asked to classify it as “false”, “true”, or “don’t know”. Responses were divided into corresponding groups: “Incorrect”, “Correct”, and “Unsure”. Associations of groups with respondent characteristics and patient reported outcomes were investigated. Responses to a further seven knowledge statements explored ampliative knowledge about hypothyroidism.

**Results:**

With regards to the principal knowledge statement, “Correct”, “Incorrect” and “Unsure” comprised 15.3%, 50.7% and 34.0% of responses to the respectively. “Incorrect” respondents were more likely than expected to live in the United Kingdom, have Hashimoto’s thyroiditis, have a recent low self-reported serum thyrotropin, be treated with liothyronine-containing medication, and use social media and the internet for hypothyroidism-related information daily. “Incorrect” responses were associated with dissatisfaction, poor perceived control of symptoms and negative impact of hypothyroidism on everyday activities. The proportion of “Incorrect” responses for seven other knowledge statements ranged between 1.8-34.9%.

**Discussion:**

Incorrect responses to the principal knowledge statement were common in this sample of people with hypothyroidism, and associated with several demographic variables and adverse patient outcomes. Our findings suggest that knowledge gaps about the significance of symptoms in relation to the diagnosis and treatment of hypothyroidism may be important in driving over-diagnosis and over-treatment. The high number of “Unsure” respondents suggests that patient education may be an effective intervention.

## 1 Introduction

Knowledge and understanding are related to information received about a topic through experience or dissemination. Unintended and deliberate propagation of false or misleading information (“misinformation” and “disinformation”, respectively) may result in false beliefs. In healthcare, misinformation that results in people holding views at odds with current evidence, can have adverse effects on patient outcomes (1).

Since 2001, thyroid hormone (TH) prescribing has been increasing (2). Most of this can be explained by over-investigation and a falling serum thyroid stimulating hormone (TSH) threshold (associated with normal TH levels) at which TH therapy is initiated (3, 4). TH treatment in patients with minor elevations of serum TSH (subclinical hypothyroidism) generally confers no benefit to quality of life (5), with a modest or no effect in cardiovascular risk (6), however this practice is widespread (2). The view that over-diagnosis and over-treatment are common is supported by a meta-analysis, showing that a third of patients on levothyroxine (L-T4) therapy, can be deprescribed (7). Medical costs of hypothyroidism in the US range between $460-$2,555 per patient per year (8). Given that approximately 10% of the US population receives treatment for hypothyroidism (9) and assuming that a third are treated inappropriately (2), the cost of over-diagnosis and over-treatment may total $0.5-2.8 billion annually. Once initiated, treatment carries a 27-41% risk of driving the serum TSH below the normal range (10–12), which is associated with increased cardiovascular morbidity (10, 13). Osteoporosis (10, 14), dementia (15, 16), and premature death (10, 13, 15, 17–20).

A likely major contributor for initiating TH replacement in the absence of biochemical evidence of hypothyroidism, or mildly elevated serum TSH, is pressure from patients on doctors in the understandable hope that unexplained symptoms will improve (21). The motivation to pursue treatment may stem from the “misconception” (a perspective contrary to best current evidence), that thyroid biochemistry is unreliable and that hypothyroidism should be diagnosed based solely on symptoms (22). This misconception is widely promoted on the internet and social media (21).

E-MPATHY (E-mode Patient self-Assessment of THYroid therapy) was a large scale international survey of patients with hypothyroidism, in which we focused on patient outcomes. We have thus far shown that patient dissatisfaction with treatment and care was associated with lack of confidence and trust with health care professionals (23), and respondent psychological traits, namely somatization (24) and Type D personality (also known as “distressed” personality, which is characterized by high levels of negative emotions and social inhibition) (25). If the above associations turn out to be causative, they will be difficult or impossible to reverse. On the other hand, patient knowledge is amenable to intervention.

Here, we explored knowledge of people treated for hypothyroidism in relation to the principal knowledge statement: “A patient with a normal thyroid blood test does not need to be treated with thyroid hormones (even if they have positive thyroid antibodies and symptoms)”, using data from E-MPATHY.

The overall aim was to explore the level of knowledge about hypothyroidism and its treatment among people treated with TH, with the following specific objectives:

1. Examine the prevalence of incorrect, correct and unsure responses in relation to eight statements about hypothyroidism, constructed by the authors.
2. Test associations between responses to the principal knowledge statement, and demographic and clinical variables.
3. Examine the contribution of demographic and clinical variables to responses to the principal knowledge statement.

## 2 Materials and methods

### 2.1 Study design

**Figure 1:**
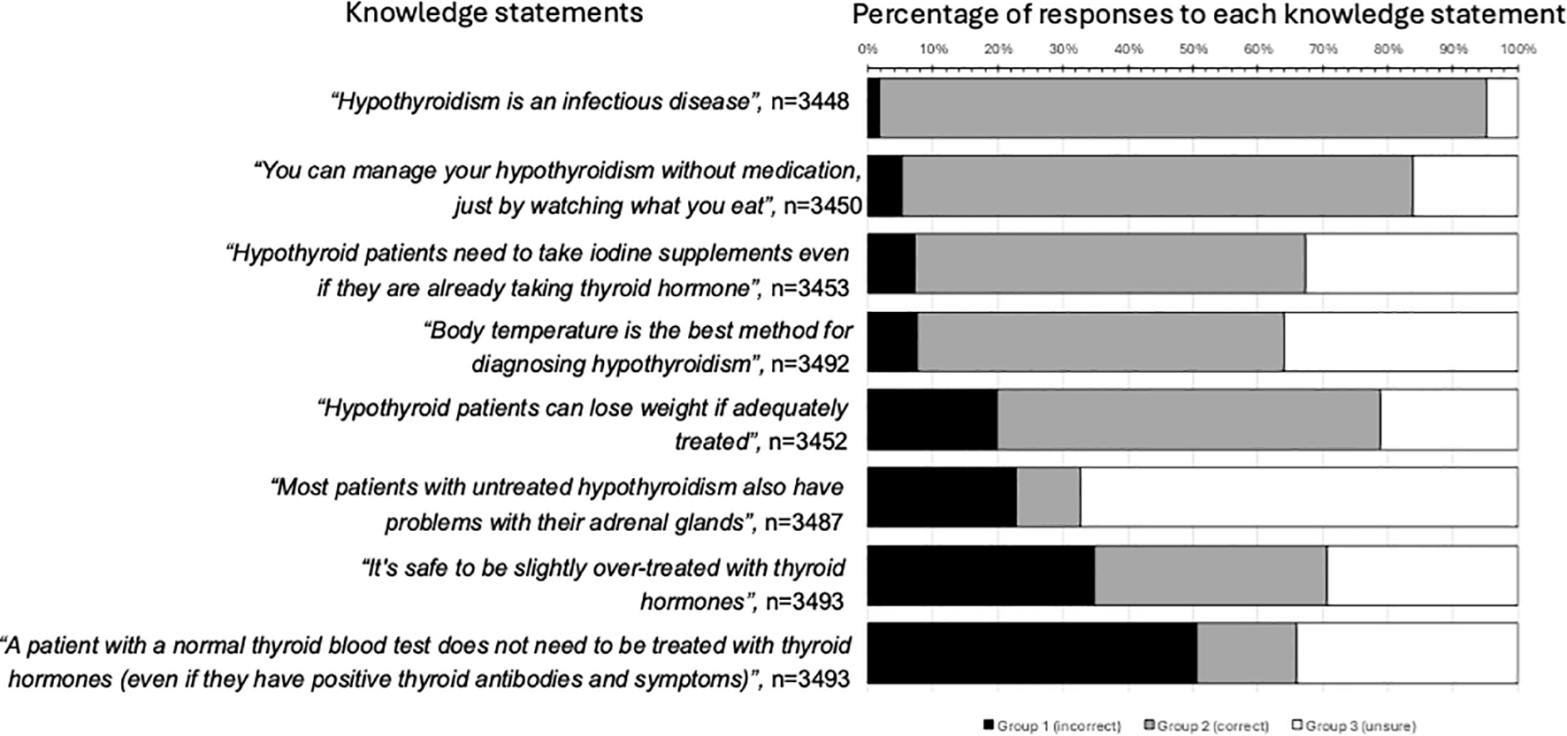
Patient responses to the eight knowledge statements. The percentage of respondents who provided incorrect answers (“Incorrect” group, black bars), correct answers (“Correct” group, gray bars) and “don’t know” answers (“Unsure” group, white bars) are shown. The knowledge statements and corresponding correct answers were: 1. A patient with a normal thyroid blood test does not need to be treated with thyroid hormones (even if they have positive thyroid antibodies and symptoms) (correct); 2. It’s safe to be slightly over-treated with thyroid hormones (e.g. having a TSH below the normal range) (incorrect); 3. Body temperature is the best method for diagnosing hypothyroidism (incorrect); 4. Most patients with untreated hypothyroidism also have problems with their adrenal glands (incorrect); 5. You can manage your hypothyroidism without medication, just by watching what you eat (incorrect); 6. Hypothyroidism is an infectious disease (incorrect); 7. Hypothyroid patients can lose weight if adequately treated (correct); 8. Hypothyroid patients need to take iodine supplements even if they are already taking thyroid hormone (incorrect).

We performed a large scale international online survey of people treated for hypothyroidism in 2020-2021 (23–25). Cognitive testing of the questionnaire with a sample of patients, followed by a pilot was used to maximize consistency of responses (23). Respondents selected “false” “true”, or “don’t know” to eight knowledge statements. Based on the “false”, “true”, or “don’t know” responses, we defined three groups: “Incorrect”, “Correct” or “Unsure” (**Figure 1**). A representative of the patient-led organization Thyroid Federation International (PL) was closely involved in designing the study and construction of the questionnaire.

### 2.2 Rationale for selection of knowledge topics

We selected and focused on the principal knowledge statement (“A patient with a normal thyroid blood test does not need to be treated with thyroid hormones (even if they have positive thyroid antibodies and symptoms))”, in advance of having access to the results because of a strong evidence-based recommendation by the American Thyroid Association (ATA) guidelines on hypothyroidism (which is widely followed in many other countries) against the use of TH in euthyroid but symptomatic people (26), and because of the associated risks and cost of over-treatment (8, 13). We included knowledge statement 2 (all knowledge statements are shown in **Figure 1**) because avoidance of over-treatment and normalization of serum TSH are the recommended targets according to ATA guidelines (26), over-treatment is common, and associations with negative health outcomes are strong (13, 17, 19). Other topics were selected because they were described by the ATA guidelines (26) as “not credible” (knowledge statement 3) or “not recommended” (knowledge statements 5 and 8). We included knowledge statement 4 because “adrenal fatigue” is commonly linked with hypothyroidism in patient internet sites, despite the prevalence of Addison’s disease in patients with Hashimoto’s thyroiditis being less than 2% (27). Knowledge statement 6 was included as a false statement because it is not regarded as a plausible etiology by experts (28), yet in one study 10% of hypothyroid patients believed that hypothyroidism can transfer to their spouse (29). We included knowledge statement 7 as this is supported by the ATA (https://www.thyroid.org/thyroid-and-weight/). Two other statements were presented to respondents on topics frequently mentioned in social media, but we did not analyse them, as there is no consensus on their veracity (“Hypothyroidism weakens the immune system” and “Untreated hypothyroidism can cause daily fluctuations of symptoms”). The categorization of “Correct” and “Incorrect” was based on the authors’ understanding and interpretation of the evidence (https://www.nice.org.uk/guidance/ng145), which we consider concordant with most thyroid experts. We recognize that the lived experiences of some patients with hypothyroidism and opinions of some thyroid experts may not align with our consensus.

During cognitive testing, participants expressed the wish to know the correct answers (as deemed by the authors) to the knowledge statements, which were made visible after completion of the questionnaire.

### 2.3 Questionnaire translations and survey platform

A text version of the questionnaire is shown in **Supplementary Data Sheet 1**. The text was translated from English to other languages (Spanish, Italian, German, French) by certified translators. Qualtrics (an online survey platform, https://www.qualtrics.com) was used. The survey took 30–45 minutes to complete and was available between November 4th 2020 and March 1st 2021.

### 2.4 Dissemination of questionnaire

Invitations were sent by e-mail to the 35 member organizations of Thyroid Federation International (https://www.thyroid-federation.org/membership/member-organizations/), who then forwarded the invitation and an attached flyer explaining the purpose of the survey in 5 languages (English, German, French, Spanish and Italian) to their individual members. In addition, the invitation was shared on Facebook and other social media.

### 2.5 Inclusion criteria

Patients were eligible if older than 18 years, and currently taking medication for hypothyroidism.

### 2.6 Institutional review board waiver statement

We followed guidance of the Danish Research Ethics Committee (https://researchethics.dk/information-for-researchers/overview-of-mandatory-reporting), Denmark being the country of the senior author (LH), which recommends that questionnaire surveys are exempt from Institutional Board Review approval. The research was completed in accordance with the Declaration of Helsinki as revised in 2013. Informed consent was submitted by respondents at the beginning of the survey.

### 2.7 Dependent variables

For objective 1 (to “examine the prevalence of incorrect, correct and unsure responses in relation to eight statements about hypothyroidism, constructed by the authors”), we recoded survey data on the eight knowledge statements (**Figure 1**). The responses were classified as: “Incorrect”, “Correct, and “Unsure”. For objectives 2, and 3, we set the dependent variable as the principal knowledge statement.

### 2.8 Independent variables

The independent variables are listed in **Supplementary Data Sheet 2**. The PHQ-15 instrument (30) for somatic symptom disorder (SSD) and the DS14 questionnaire for type D personality (31) were embedded in the questionnaire. The question about use of social media and the internet related specifically to information about hypothyroidism (“to what extent do you use social media and the internet to find out information about your hypothyroidism?”, **Supplementary Data Sheet 1**).

### 2.9 Statistical methods

We used Python 3.11, via Spyder 4.5.3, for our analyses.

#### 2.9.1 Descriptive statistics

For each knowledge statement dependent variable (**Figure 1**), we calculated the percentage of respondents in the groups “Incorrect”, “Correct”, and “Unsure”.

#### 2.9.2 Chi-square tests

We examined associations between the dependent variable (principal knowledge statement) and the independent variables via chi-squared tests, with a null hypothesis of no association. We applied a Bonferroni correction to mitigate multiple testing of the same outcome (adjusted test threshold α = 0.05/23 = 0.0022). To assess directionality associations, we used differences between observed and statistically expected distributions.

#### 2.9.3 Gradient boosting decision tree modelling

We used a Decision Tree approach, and developed a Gradient Boosting Decision Tree (GBDT) model to examine the impact of the independent variables on the principal knowledge statement. GBDT modelling is a machine learning “ensemble technique” (32) that creates a combined classification of the dataset to explore the importance of independent variables (“features”) in predicting an outcome (33). GBDT combines multiple decision trees to classify data and assess the importance of predictors. These trees split the dataset into sub-groups to maximize within-group similarity, improving prediction accuracy by minimizing a log-loss function (34). The LightGBM package was used due to its efficiency with multi-category outcomes and multiple categorical predictors (34, 35) (https://lightgbm.readthedocs.io/en/latest/Python-Intro.html).

To mitigate overfitting, we split the data into training and testing sets and optimized hyperparameters (e.g., gradient method, maximum number of leaves, minimum tree depth) using the Hyperopt package (https://hyperopt.github.io/hyperopt/) (36). The model trained with these optimized parameters used an early stopping rule after 100 iterations without improvement. Performance was evaluated using training versus testing plots, feature importance plots, confusion matrices, and derived metrics.

## 3 Results

In total, 3915 survey responses were received (**Table 1**). Of these 89.2% (3493/3915) provided a valid response to the principal knowledge statement and 87.4% (3421/3915) to all eight statements. The response rate cannot be calculated as the dissemination of the questionnaire was via a variety of patient networks and social media, and therefore the number of eligible patients was unknown.

**Table 1:** Demographic and clinical characteristics of the study population.

	% (n)
DEMOGRAPHIC CHRACTERISTICS	
Gender	
Female	94.4 (3298/3493)
Male	4.7 (164/3493)
Prefer not to say/Prefer to self-identify	0.8 (29/3493)
Missing data	0.1 (2/3493)
Age	
18-50	52.1 (1821/3493)
>51	45.7 (1595/3493)
Missing data	2.2 (77/3493)
Marital status	
Married/partnership	68.7 (2398/3493)
Single/divorced/widowed/Prefer not to say/Other	29.0 (1012/3493)
Missing data	2.4 (83/3493)
Employment status	
Working (full time, part-time, student, carer)	74.9 (2615/3493)
Not working	17.2 (602/3493)
Prefer not to say/Other	5.6 (195/3493)
Missing data	2.3 (81/3493)
Ethnic background	
White	90.0 (3145/3493)
Other/Prefer not to	7.6 (267/3493)
Missing data	2.3 (81/3493)
Countries (top five with highest participants)	
UK	35.8 (1250/3493)
France	17.1 (596/3493)
Sweden	5.5 (191/3493)
Finland	4.2 (146/3493)
Australia	4.0 (140/3493)
Other^1^	29.2 (1021/3493)
Missing data	4.3 (149/3493)
Years in education	
8 years or less	7.7 (269/3493)
9–16 years	41.7 (1455/3493)
More than 16 years	46.1 (1612/3493)
Prefer not to say	2.2 (77/3493)
Missing data	2.3 (80/3493)
Household income	
Above average	31.0 (1082/3493)
Average	43.8 (1531/3493)
Below average	18.1 (632/3493)
Prefer not to say/Don’t know	4.8 (168/3493)
Missing data	2.3 (80/3493)
Number of comorbidities	
No comorbid conditions	16.3 (571/3493)
One	25.3 (885/3493)
Two or more	47.9 (1672/3493)
Missing data	10.4 (365/3493)
Use of social media and internet for information on hypothyroidism	
Never	11.5 (400/3493)
Less than once a month	32.4 (1131/3493)
Once a month	18.3 (638/3493)
Once or twice a week	21.7 (758/3493)
Daily	14.1 (492/3493)
Missing data	2.1 (74/3493)
Probable Somatic Symptom Disorder	
Yes	56.4 (1969/3493)
No	40.0 (1398/3493)
Missing data	3.6 (126/3493)
Type D personality	
Yes	53.9 (1884/3493)
No	45.8 (1601/3493)
Missing data	0.2 (8/3493)
Anxiety	
Yes	65.2 (2279/3493)
No	33.1 (1157/3493)
Missing data	1.6 (57/3493)
Low mood/depression	
Yes	67.8 (2367/3493)
No	30.7 (1072/3493)
Missing data	1.5 (54/3493)
DISEASE CHARACTERISTICS	
Cause of hypothyroidism	
Hashimoto/autoimmune disease	36.5 (1276/3493)
Treatment for Graves’ disease or hyperthyroidism	8.2 (287/3493)
Treatment for thyroid cancer	12.9 (450/3493)
Treatment for benign goiter	5.0 (173/3493)
Pregnancy related	3.8 (132/3493)
Other	33.5 (1171/3493)
Missing data	0.1 (4/3493)
Duration of hypothyroidism (years)	
<2	10.0 (350/3493)
2-10	36.3 (1267/3493)
>10	51.6 (1801/3493)
Missing data	2.1 (75/3493)
Highest ever recorded serum TSH (mU/L)*	
<4.0	10.6 (369/3493)
4.0-10.0	24.2 (847/3493)
>10.0	32.8 (1144/3493)
Missing data	32.4 (1133/3493)
Current treatment for hypothyroidism	
L-T4	75.0 (2621/3493)
L-T4 + L-T3	10.1 (353/3493)
DTE	7.6 (265/3493)
L-T3	2.1 (72/3493)
Missing data	5.2 (182/3493)
Most recent serum TSH (mU/L)*	
<0.1	14.6 (509/3493)
0.1-<0.4	16.1 (562/3493)
0.4-<4.0	35.0 (1222/3493)
4.0-10.0	8.0 (280/3493)
>10.0	3.5 (124/3493)
Missing TSH data	22.8 (796/3493)
PATIENT REPORTED OUTCOMES	
Hypothyroidism symptom control with medication^2^	
Controlled	41.3 (1442/3493)
Not controlled	53.3 (1862/3493)
Missing data	5.4 (189/3493)
Confidence and trust in healthcare staff^3^	
Has confidence and trust	53.3 (1863/3493)
No confidence or trust	20.0 (698/3493)
Missing data	26.7 (932/3493)
Satisfaction with overall care and treatment for hypothyroidism^4^	
Satisfied	36.5 (1274/3493)
Not satisfied	62.5 (2183/3493)
Missing data	1.0 (36/3493)
Impact of hypothyroidism on everyday activities^5^	
Impacted	79.1 (2764/3493)
Not impacted	15.8 (553/3493)
Missing data	4.5 (176/3493)

^1^ “Other” countries: Afghanistan, Albania, Algeria, Argentina, Austria, Belgium, Bolivia, Brazil, Central African Republic, Canada, Chilli, Colombia, Costa Rica, Croatia, Czech Republic, Cyprus, Denmark, Ecuador, Estonia, Ethiopia, Georgia, Germany, Greece, Grenada, Honduras, Hungary, India, Ireland, Israel, Italy, Japan, Jordan, Kenya, Luxemburg, Madagascar, Malaysia, Malta, Mexico, Morocco, Netherlands, New Zealand, Nigeria, Norway, Paraguay, Peru, Philippines, Poland, Portugal, Romania, Slovenia, South Africa, Spain, Switzerland, Thailand, Trinidad and Tobago, Tunisia, Turkey, United Arab Emirates, United States of America, Uruguay, Venezuela, Vietnam.

^2^ “Strongly agree” and “tend to agree” were coded as “controlled”. “Strongly disagree” and “tend to disagree”, and “neither agree nor disagree” were coded as “not controlled”.

^3^ “Yes always” and “Yes to some extent” were coded as “has confidence and trust”; “no” was coded as “no confidence or trust”.

^4^ “Very satisfied” and “slightly satisfied” were coded as “satisfied”; “very dissatisfied”, “slightly dissatisfied”, and “neither satisfied nor dissatisfied” were coded as “not satisfied”.

^5^ “Strongly disagree” and “tend to disagree” were coded as “not impacted”; “strongly agree”, “tend to agree” and “neither agree nor disagree” were coded as “impacted”.

*The TSH values were self-reported.

### 3.1 Prevalence of incorrect responses about hypothyroidism and its treatment

Half of the respondents regarded the principal knowledge statement (“A patient with a normal thyroid blood test does not need to be treated with thyroid hormones (even if they have positive thyroid antibodies and symptoms)”, as false (50.7%, 1771/3493, “Incorrect” group), consistent with the false belief that symptomatic individuals merit treatment with thyroid hormones even if their thyroid blood tests are normal. The same knowledge statement was described as true by 15.3% (533/3493) of respondents (“Correct” group), while 34.0% (1189/3493) were in the “Unsure” group. The levels of incorrect responses among the eight knowledge statements varied between 1.8% (61/3448) to 50.7% (1771/3493) (**Figure 1**). Very few respondents classified all statements correctly (1.1%, 38/3421) (**Supplementary Data Sheet 3**), or all incorrectly 1.0% (35/3421). The modal correct number was four (25.9%, 886/3421).

### 3.2 Associations between responses to the principal knowledge statement, and demographic and other baseline variables

**Figure 2:**
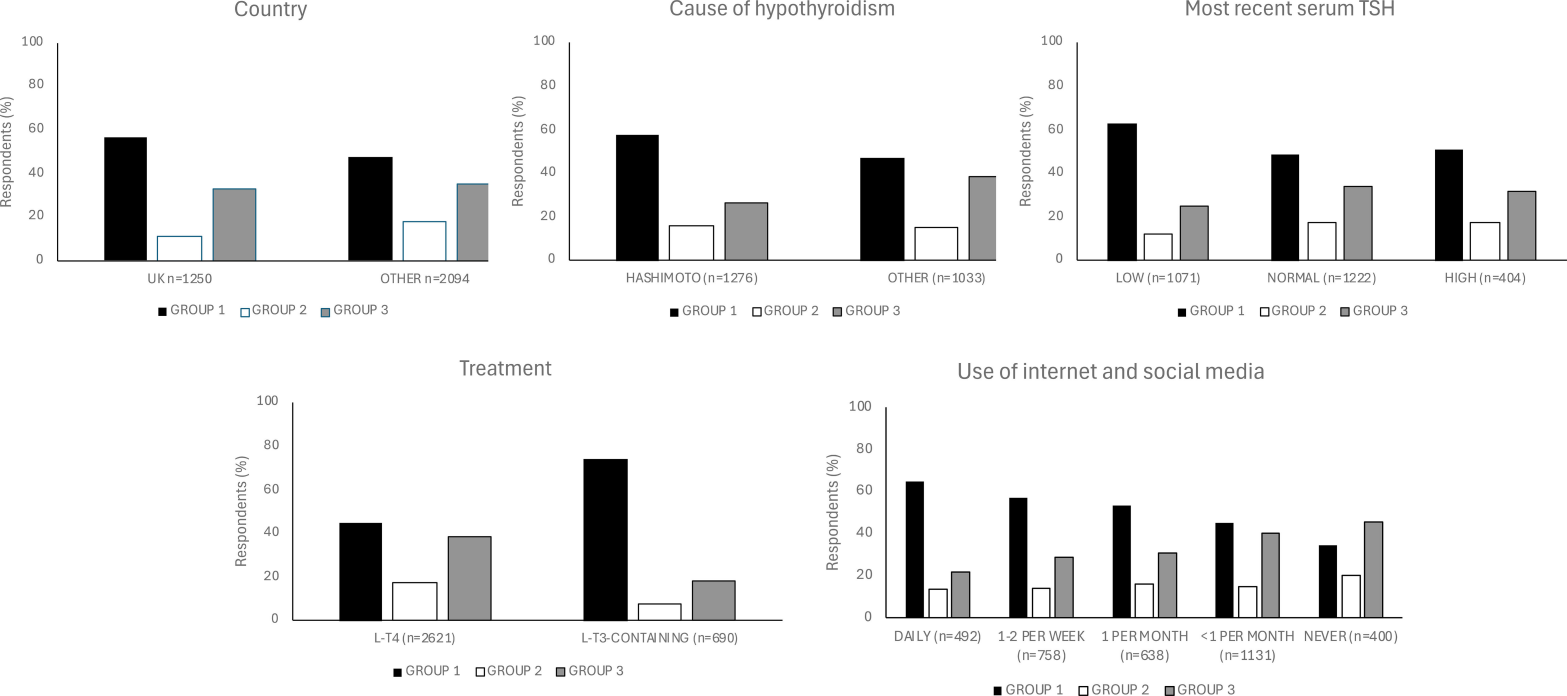
Responses to a principal knowledge statement (“A patient with a normal thyroid blood test does not need to be treated with thyroid hormones (even if they have positive thyroid antibodies and symptoms)”) and statistically significant associations with demographic and baseline characteristics. Responses were categorized into three groups: “Incorrect”, “Correct” and “Unsure”. To calculate the percentages, we determined the number of respondents with each demographic or baseline characteristic (e.g., UK residence) within each group (e.g., “Incorrect” group). These numbers were then divided by the total number of respondents across all three groups for that characteristic, excluding any missing data. The P values for associations between the “Incorrect” group and the variables “country”, “cause of hypothyroidism”, “most recent TSH”, “treatment for hypothyroidism” and “use of internet and social media” using Bonferroni adjustment, were <0.001 (**Supplementary Data Sheet 4**). L-T4, levothyroxine; L-T3, liothyronine; DTE, desiccated thyroid extract.

Statistically significant associations are shown in **Figure 2** and **Supplementarys Data Sheets 4, 5**. Compared to the null hypothesis of no association between the variables, we found more respondents than expected: were from France and were in the “Correct” group; had Hashimoto’s as cause and were in the “Incorrect” group; had a recent self-reported TSH <0.1 mU/L and were in the “Incorrect” group; were treated with desiccated thyroid extract (DTE) and combination of L-T4 with liothyronine (L-T3) and were in the “Incorrect” group; used social media and the internet for hypothyroidism-related information daily and were in the “Incorrect” group, and never used social media and the internet for hypothyroidism-related information and were in the “Correct” and “Unsure” groups.

### 3.3 Associations between responses to the principal knowledge statement, and patient outcome variables

**Figure 3:**
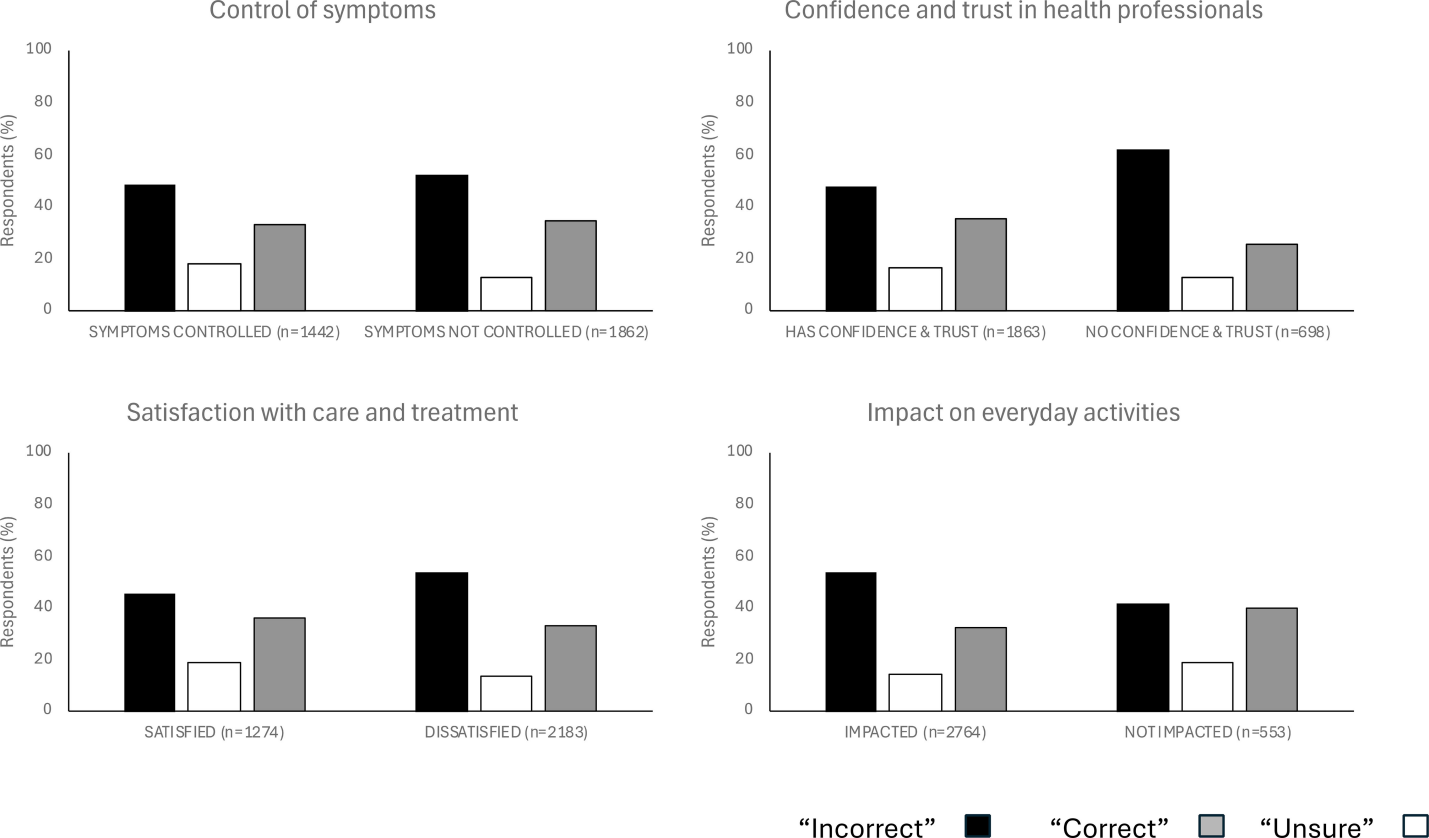
Responses to the principal knowledge statement (“A patient with a normal thyroid blood test does not need to be treated with thyroid hormones (even if they have positive thyroid antibodies and symptoms)”) and statistically significant associations with patient outcomes. The responses were categorized into three groups: “Incorrect”, “Correct” and “Unsure”. To calculate the percentages, we determined the number of respondents with each patient outcome (e.g., control of symptoms) within each group (e.g., “Incorrect” group). These numbers were then divided by the total number of respondents across all three groups for that patient outcome, excluding any missing data. The P values for associations between the “Incorrect” group and the variables “control of symptoms”, “confidence and trust in health professionals”, “satisfaction with care and treatment”, and “treatment on everyday activities” using Bonferroni adjustment, were <0.001 (**Supplementary Data Sheet 4**).

Statistically significant associations are shown in **Figure 3**. Compared to the null hypothesis of no association between the variables, we found that more respondents than statistically expected, stated that their symptoms were controlled by their thyroid medication and were in the “Correct” group; had no confidence and trust in healthcare staff and were in the “Incorrect” group; were satisfied with care and treatment and were in “Correct” group; were not impacted in daily living and were in “Correct” and “Unsure” groups.

### 3.4 Contribution of demographic and clinical variables to responses to the principal knowledge statement

**Figure 4:**
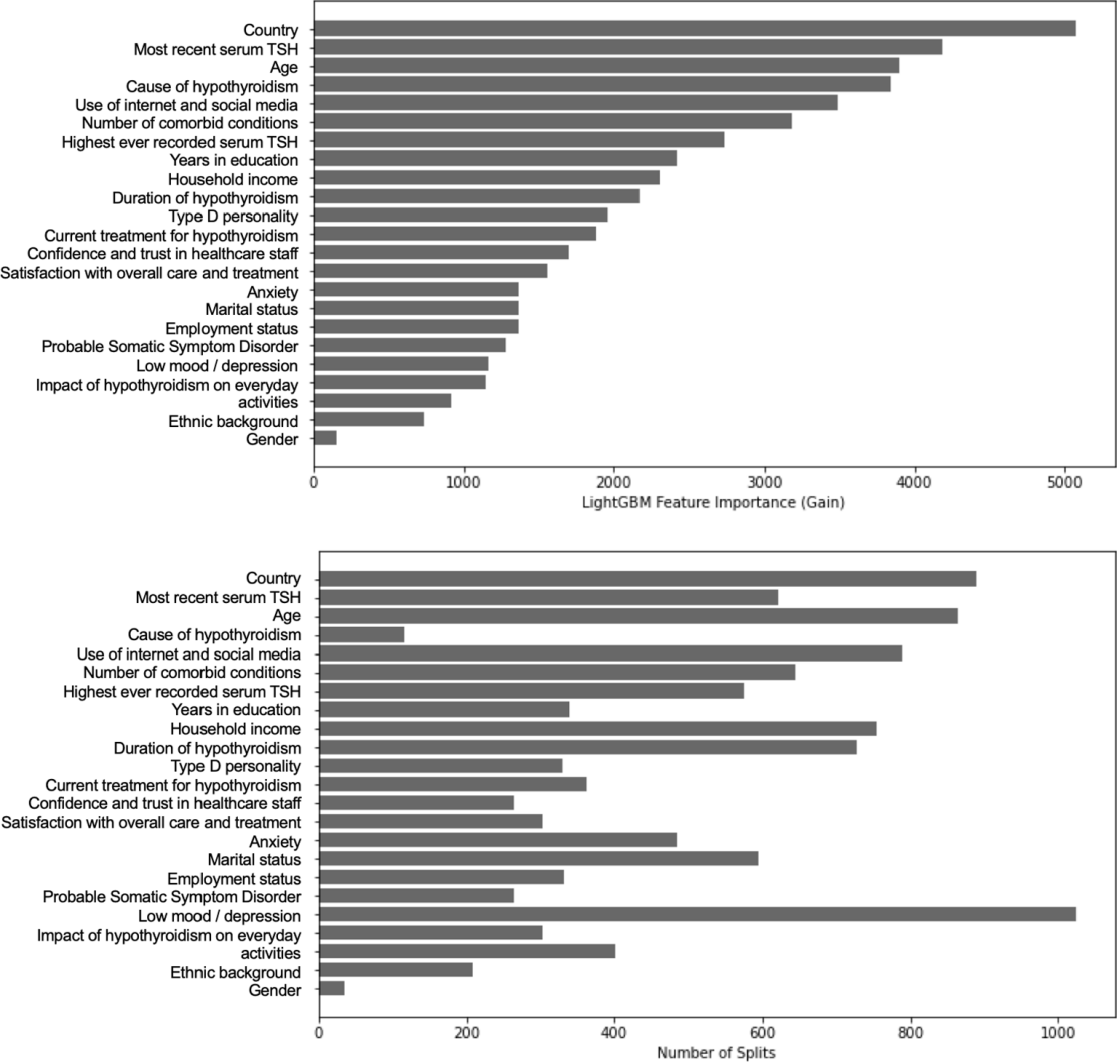
Feature importance plots from a Gradient Boosting Decision Tree (GBDT) model, predicting the “group” (“Incorrect”, “Correct” or “Unsure”) of the principal knowledge statement (“A patient with a normal thyroid blood test does not need to be treated with thyroid hormones (even if they have positive thyroid antibodies and symptoms)”). Plot A (Gain Importance) shows how much each feature improves model accuracy, whilst Plot B (Feature Frequency) shows how often each feature is used in decision splits. Country, Use of Internet and Social Media, recent TSH level, Treatment type, and Age were the strongest predictors.

The final ensemble GBDT model (held in computing memory) incorporated 315 component decision trees (**Supplementary Data Sheet 6**). Feature importance in this model was assessed using “gain importance,” which reflects the contribution of each feature to reducing overall model loss. The top five features by gain were: country, use of social media and the internet for hypothyroidism-related information, recent self-reported TSH, treatment type for hypothyroidism, and age (**Figure 4**). The selection of age as an important feature by GBDT, despite not emerging in simpler two-factor tests, suggests a more complex relationship between age and the principal knowledge statement. Although not significant in bivariate testing, the relationship between age and response category was non-linear: respondents aged 51–60 were over-represented in the “Incorrect” category, while those over 60 were under-represented in that group. The “Unsure” category was less frequent among those aged 51–60.

GBDT models are evaluated based on predictive performance metrics rather than statistical significance. The key metrics for this model included an overall accuracy of 55.3%, weighted average precision of 49.8%, recall of 55.3%, and F1-score of 49.8%. The Area Under the Curve (AUC) was 0.65, indicating moderate discriminative ability. Performance varied across response groups, with the model better classifying “Incorrect” responses (F1 = 0.67, recall = 81.7%) compared to “Correct” (F1 = 0.03, recall = 1.6%) and “Unsure” (F1 = 0.36, recall = 41.3%). These results suggest that while the model identified patterns in the data, predictive performance was limited, likely due to confounding or unaccounted variability.

Unlike traditional statistical models, GBDT does not rely on P values or hypothesis testing and instead optimizes predictive accuracy by iteratively reducing “loss”. While the model provides insights into feature contributions, its moderate performance metrics indicate that findings should be interpreted cautiously, and further investigation is needed to clarify underlying relationships (**Supplementary Data Sheet 7**).

### 3.5 Other knowledge statements

The likelihood of “Incorrect” respondents to the principal knowledge statement responding incorrectly to one, two or three additional knowledge statements was 71.2% (1242/1742), 35.0% (610/1742) and 12.1% (210/1742), respectively. The likelihood of “Incorrect” respondents to the principal knowledge statement selecting incorrect answers to the other knowledge statements ranged from 1.9% (34/1751) for “hypothyroidism being an infectious disease”, to 46.2% (818/1769) for “over-treatment with thyroid hormones being safe” (**Supplementary Data Sheet 8**).

A significant minority (14.6%, 509/3493) of respondents had a recent self-reported serum TSH of less than 0.1 mU/L (**Table 1**), and most of these (65.0%, 331/509) expressed the view that over-treatment with thyroid hormones is safe (this statistically significant association appears to be driven by difference in observed and expected frequencies for low recent self-reported serum TSH and “Incorrect” responses, as indicated by the large chi partial of 130). Responses to this question were no different among patients with thyroid cancer and the rest of respondents.

## 4 Discussion

Dissatisfaction among people treated for hypothyroidism is common, and drives patients to seek treatments of questionable value that may be harmful (37, 38). Our data provide new insights and understanding on the possible contribution of holding a view at odds with current evidence.

A major finding was that half of respondents indicated that hypothyroidism can be diagnosed and treated based on symptoms, even when the biochemistry is normal. This probably relates to two main contributors. First, a debate has been ongoing among experts on the definition of the reference range for serum TSH (39–41). This in itself indicates that the answer is not clear-cut, and awareness of this discourse may lead non-experts to conclude that thyroid function tests are unreliable and untrustworthy. Second, the popularity in social media and patient websites of the so called “Wilson’s syndrome”, which claims that the presence of common and nonspecific symptoms, and relatively low body temperature define hypothyroidism (https://www.thyroid.org/american-thyroid-association-statement-on-wilsons-syndrome/). The above, combined with the third of respondents who agreed with the statement that “It’s safe to be slightly over-treated with thyroid hormones (e.g. having a TSH below the normal range)”, are relevant to the concerning trend noted of a falling serum TSH threshold for initiation of treatment for hypothyroidism and the rising number of euthyroid people treated with TH (2, 42, 43). The prevalence of “Incorrect” responses that can potentially impact on treatment choices (knowledge statements 1–5 and 8) varied substantially (between 1.8-50.7%). It is unclear why that was the case, but may relate to the diverse backgrounds of participants.

Approximately a third of respondents were unsure about the answer to the principal knowledge statement. This is encouraging, as those who are uncertain may be more willing to accept the current scientific consensus than those who hold firm false views (44).

The association noted in this study between lack of knowledge and dissatisfaction with treatment and lack of confidence and trust in healthcare professionals, suggests that incorrect beliefs may undermine the hypothyroid patient-physician relationship and impact negatively on the patient experience (45). Conversely, lack of confidence and trust in healthcare professionals may drive patients to seek information elsewhere and expose them to misinformation. Therefore, such patients may benefit from additional time by health professionals to address lack of knowledge. The association with use of social media and the internet for hypothyroidism-related information, indicates that these may be important sources of misinformation, in accordance with other studies (46), although for many respondents lack of knowledge may be the principal reason that they chose incorrect answers. Our data showed that fewer respondents than expected were unsure about the answer to the principal knowledge statement, if usage of social media and the internet for hypothyroidism-related information was daily. This suggests that people treated for hypothyroidism trust the information they obtain from internet sources and that making scientifically sound internet content more accessible to patients is a worthy objective (47).

The associations between “Incorrect” responses to the principal knowledge statement with low recent self-reported serum TSH is in keeping with the concept that such patients are likely to underestimate the risks of over-treatment. The UK emerged as highest ranked country for “Incorrect” responses to the principal knowledge statement, which was unexpected, as misinformation about other health topics (e.g. vaccination) is highest in countries with poor socioeconomic backgrounds (48). Possible explanations include high social media consumption (https://datareportal.com/), and the fact that the UK population has one of the highest levels of distrust in authorities in the world (https://www.statista.com/statistics/1362804/trust-government-world/),

The final GBDT model achieved an AUC of less than 0.8 (a threshold typically considered sufficient for clinical applications); this highlights the complexity of predicting cognitive phenomena, using demographic and sociological variables. The model showed some ability to recall instances of “Incorrect” responses, but specificity was generally low across groups. These limitations could be due to unobserved confounding factors and the inherent difficulty in capturing the nuanced processes by which individuals form beliefs. Therefore, the results should be interpreted as exploratory and hypothesis-generating, rather than as definitive predictive tools.

Limitations include inability to calculate the response rate due to the mode of survey administration, lack of information on the characteristics of non-responders, use of self-reported data that were not validated independently and over-representation of some countries. The participants in our study therefore may not be representative of the entire population of treated patients with hypothyroidism. The survey was conducted during the COVID pandemic and it is possible that it impacted on the number and content of responses. The survey was only offered online and only in five languages, which will have resulted in limited accessibility. Although the demographic characteristics of participants in our study match those reported in the literature, dissatisfied patients are more likely to respond to surveys and this group was probably over-represented. The principal knowledge question did not specify whether it related to primary or secondary hypothyroidism, and in the latter scenario a normal serum TSH is misleading. In mitigation of the above shortcomings, cognitive testing of the questionnaire, followed by a pilot maximized consistency of responses. The questionnaire specifically encouraged respondents to provide answers based on respondents’ typical experience, not based on those that could be attributed to the pandemic (**Supplementary Data Sheet 1**). The sample size was large from multiple countries and cultural backgrounds. Participation of a patient representative in our research team (PL) ensured that the patient perspective was included in the design of the study and interpretation of the data.

Treatment of euthyroid people with thyroid hormones is a widespread and worrying phenomenon (3, 4, 7). Ultimately, prescribers bear the responsibility for this non-evidence based practice (49–53). Whether prescribers of thyroid hormones in our cohort were primary care physicians or endocrinologists is unknown. Our findings provide new evidence that incorrect patient beliefs may be important drivers for over-diagnosis of hypothyroidism and over-treatment with thyroid hormones and for patient dissatisfaction with treatment and care.

In conclusion, incorrect responses were common in this sample of people with hypothyroidism, and associated with several demographic variables and adverse patient outcomes. Our findings suggest that knowledge gaps about the significance of symptoms in relation to the diagnosis and treatment of hypothyroidism may be important in driving over-diagnosis and over-treatment. The high number of “Unsure” respondents suggests that patient education may be an effective intervention.

The survey sheds light on the disparity between patient and clinician perspective and further research should explore how these can be realigned.

## Data Availability

The raw data supporting the conclusions of this article will be made available by the authors, without undue reservation.

## References

[1] van der Linden S. Misinformation: susceptibility, spread, and interventions to immunize the public. Nat Med. (2022) 28:460–7. doi: 10.1038/s41591-022-01713-6, PMID: 35273402

[2] Brito JP Ross JS El Kawkgi OM Maraka S Deng Y Shah ND. Levothyroxine use in the United States, 2008-2018. JAMA Intern Med. (2021) 181:1402–5. doi: 10.1001/jamainternmed.2021.2686, PMID: 34152370 PMC8218227

[3] Esfandiari NH Papaleontiou M. Levothyroxine prescribing: why simple is so complex. J Clin Endocrinol Metab. (2024) 109:e1406–e7. doi: 10.1210/clinem/dgad585, PMID: 37793166

[4] Ayala IN Soto Jacome C Toro-Tobon D Golembiewski E Garcia-Bautista A Hidalgo J. Appropriateness of levothyroxine prescription: a multicenter retrospective study. J Clin Endocrinol Metab. (2024) 109:e765–e72. doi: 10.1210/clinem/dgad517, PMID: 37656124 PMC10795923

[5] Hegedüs L Bianco AC Jonklaas J Pearce SH Weetman AP Perros P. Primary hypothyroidism and quality of life. Nat Rev Endocrinol. (2022) 18:230–42. doi: 10.1038/s41574-021-00625-8, PMID: 35042968 PMC8930682

[6] Yu OHY Filliter C Filion KB Platt RW Grad R Renoux C. Levothyroxine treatment of subclinical hypothyroidism and the risk of adverse cardiovascular events. Thyroid. (2024) 34:1214–24. doi: 10.1089/thy.2024.0227, PMID: 39104265

[7] Burgos N Toloza FJK Singh Ospina NM Brito JP Salloum RG Hassett LC. Clinical outcomes after discontinuation of thyroid hormone replacement: a systematic review and meta-analysis. Thyroid. (2021) 31:740–51. doi: 10.1089/thy.2020.0679, PMID: 33161885 PMC8110016

[8] Hepp Z Lage MJ Espaillat R Gossain VV. The direct and indirect economic burden of hypothyroidism in the United States: a retrospective claims database study. J Med Econ. (2021) 24:440–6. doi: 10.1080/13696998.2021.1900202, PMID: 33685322

[9] Wyne KL Nair L Schneiderman CP Pinsky B Antunez Flores O Guo D. Hypothyroidism Prevalence in the United States: A Retrospective study combining national health and nutrition examination survey and claims data, 2009-2019. J Endocr Soc. (2022) 7:bvac172. doi: 10.1210/jendso/bvac172, PMID: 36466005 PMC9706417

[10] Flynn RW Bonellie SR Jung RT MacDonald TM Morris AD Leese GP. Serum thyroid-stimulating hormone concentration and morbidity from cardiovascular disease and fractures in patients on long-term thyroxine therapy. J Clin Endocrinol Metab. (2010) 95:186–93. doi: 10.1210/jc.2009-1625, PMID: 19906785

[11] Somwaru LL Arnold AM Joshi N Fried LP Cappola AR. High frequency of and factors associated with thyroid hormone over-replacement and under-replacement in men and women aged 65 and over. J Clin Endocrinol Metab. (2009) 94:1342–5. doi: 10.1210/jc.2008-1696, PMID: 19126628 PMC2682480

[12] Okosieme OE Belludi G Spittle K Kadiyala R Richards J. Adequacy of thyroid hormone replacement in a general population. QJM. (2011) 104:395–401. doi: 10.1093/qjmed/hcq222, PMID: 21109503

[13] Lillevang-Johansen M Abrahamsen B Jorgensen HL Brix TH Hegedüs L. Duration of over- and under-treatment of hypothyroidism is associated with increased cardiovascular risk. Eur J Endocrinol. (2019) 180:407–16. doi: 10.1530/EJE-19-0006, PMID: 31035256

[14] Abrahamsen B Jorgensen HL Laulund AS Nybo M Bauer DC Brix TH. The excess risk of major osteoporotic fractures in hypothyroidism is driven by cumulative hyperthyroid as opposed to hypothyroid time: an observational register-based time-resolved cohort analysis. J Bone Miner Res. (2015) 30:898–905. doi: 10.1002/jbmr.2416, PMID: 25431028

[15] Folkestad L Brandt F Lillevang-Johansen M Brix TH Hegedüs L. Graves’ disease and toxic nodular goiter, aggravated by duration of hyperthyroidism, are associated with Alzheimer’s and vascular dementia: a registry-based long-term follow-up of two large cohorts. Thyroid. (2020) 30:672–80. doi: 10.1089/thy.2019.0672, PMID: 31984866

[16] Thvilum M Brandt F Lillevang-Johansen M Folkestad L Brix TH Hegedüs L. Increased risk of dementia in hypothyroidism: A Danish nationwide register-based study. Clin Endocrinol (Oxf). (2021) 94:1017–24. doi: 10.1111/cen.14424, PMID: 33484007

[17] Evron JM Hummel SL Reyes-Gastelum D Haymart MR Banerjee M Papaleontiou M. Association of thyroid hormone treatment intensity with cardiovascular mortality among us veterans. JAMA Netw Open. (2022) 5:e2211863. doi: 10.1001/jamanetworkopen.2022.11863, PMID: 35552725 PMC9099430

[18] Lillevang-Johansen M Abrahamsen B Jorgensen HL Brix TH Hegedüs L. Over- and under-treatment of hypothyroidism is associated with excess mortality: a register-based cohort study. Thyroid. (2018) 28:566–74. doi: 10.1089/thy.2017.0517, PMID: 29631518

[19] Thayakaran R Adderley NJ Sainsbury C Torlinska B Boelaert K Sumilo D. Thyroid replacement therapy, thyroid stimulating hormone concentrations, and long term health outcomes in patients with hypothyroidism: longitudinal study. BMJ. (2019) 366:l4892. doi: 10.1136/bmj.l4892, PMID: 31481394 PMC6719286

[20] Abrahamsen B Jorgensen HL Laulund AS Nybo M Brix TH Hegedüs L. Low serum thyrotropin level and duration of suppression as a predictor of major osteoporotic fractures-the OPENTHYRO register cohort. J Bone Miner Res. (2014) 29:2040–50. doi: 10.1002/jbmr.2244, PMID: 24723381

[21] Hegedüs L van der Feltz-Cornelis CM Papini E Nagy EV Weetman AP Perros P. Medically not yet explained symptoms in hypothyroidism. Nat Rev Endocrinol. (2024) 20:685–93. doi: 10.1038/s41574-024-01022-7, PMID: 39138377

[22] Tang C Garber J. Wilson’s syndrome (Low T3 syndrome)”. In: McDermott MT, editor. Management of Patients with Pseudo-Endocrine Disorders: A Case-Based Pocket Guide. Springer, Cham (2019). p. 273–89.

[23] Perros P Hegedüs L Nagy EV Papini E Hay HA Abad-Madronero J. The impact of hypothyroidism on satisfaction with care and treatment and everyday living: results from e-mode patient self-assessment of thyroid therapy, a cross-sectional, international online patient survey. Thyroid. (2022) 32:1158–68. doi: 10.1089/thy.2022.0324, PMID: 35959734

[24] Perros P Nagy EV Papini E van der Feltz-Cornelis CM Weetman AP Hay HA. Hypothyroidism and somatization: results from e-mode patient self-assessment of thyroid therapy, a cross-sectional, international online patient survey. Thyroid. (2023) 33:927–39. doi: 10.1089/thy.2022.0641, PMID: 37134204

[25] Perros P Nagy EV Papini E Abad-Madronero J Lakwijk P Poots AJ. Hypothyroidism and type d personality: results from E-MPATHY, a Cross-sectional International Online Patient Survey. J Clin Endocrinol Metab. (2024) 110:e97–e108. doi: 10.1210/clinem/dgae140, PMID: 38591918 PMC11651697

[26] Jonklaas J Bianco AC Bauer AJ Burman KD Cappola AR Celi FS. Guidelines for the treatment of hypothyroidism: prepared by the american thyroid association task force on thyroid hormone replacement. Thyroid. (2014) 24:1670–751. doi: 10.1089/thy.2014.0028, PMID: 25266247 PMC4267409

[27] Boelaert K Newby PR Simmonds MJ Holder RL Carr-Smith JD Heward JM. Prevalence and relative risk of other autoimmune diseases in subjects with autoimmune thyroid disease. Am J Med. (2010) 123:183.e1–e9. doi: 10.1016/j.amjmed.2009.06.030, PMID: 20103030

[28] Taylor PN Medici MM Hubalewska-Dydejczyk A Boelaert K. Hypothyroidism. Lancet. (2024) 404:1347–64. doi: 10.1016/S0140-6736(24)01614-3, PMID: 39368843

[29] Goel A Shivaprasad C Kolly A Pulikkal AA Boppana R Dwarakanath CS. Frequent occurrence of faulty practices, misconceptions and lack of knowledge among hypothyroid patients. J Clin Diagn Res. (2017) 11:OC15–20. doi: 10.7860/JCDR/2017/29470.10196, PMID: 28892955 PMC5583790

[30] Kroenke K Spitzer RL Williams JB. The PHQ-15: validity of a new measure for evaluating the severity of somatic symptoms. Psychosom Med. (2002) 64:258–66. doi: 10.1097/00006842-200203000-00008, PMID: 11914441

[31] Denollet J. DS14: standard assessment of negative affectivity, social inhibition, and Type D personality. Psychosom Med. (2005) 67:89–97. doi: 10.1097/01.psy.0000149256.81953.49, PMID: 15673629

[32] Sagi O Rokach L. Ensemble learning: A survey. Wiley Interdiscip Rev Data Min Knowl Discov. (2018) 8:e1249. doi: 10.1002/widm.1249

[33] Seto H Oyama A Kitora S Toki H Yamamoto R Kotoku J. Gradient boosting decision tree becomes more reliable than logistic regression in predicting probability for diabetes with big data. Sci Rep. (2022) 12:15889. doi: 10.1038/s41598-022-20149-z, PMID: 36220875 PMC9553945

[34] Friedman J. Greedy function approximation: A gradient boosting machine. Ann Statistics. (2001) 29:1189–232. doi: 10.1214/aos/1013203451

[35] Ke G Meng Q Finley T Wang T Chen W Ma W. LightGBM: A highly efficient gradient boosting decision tree. Advances in neural information processing systems. In: Guyon I Luxburg UV Bengio S Wallach H Fergus R Vishwanathan S Garnett R, editors. Advances in Neural Information Processing Systems 30 (NIPS 2017). Long Beach, CA, USA: Curran Associates, Inc (2017). p. 3147–55.

[36] Bergstra J Yamins D Cox D. Hyperopt: A python library for optimizing the hyperparameters of machine learning algorithms. Proc 12th Python Sci Conf. (2013) 13:20. doi: 10.25080/Majora-8b375195-003

[37] Perros P van der Feltz-Cornelis C Papini E Nagy EV Weetman AP Hegedüs L. The enigma of persistent symptoms in hypothyroid patients treated with levothyroxine: A narrative review. Clin Endocrinol (Oxf). (2023) 98:461–8. doi: 10.1111/cen.14473, PMID: 33783849

[38] Hegedüs L Nagy EV Papini E Perros P. Limiting the use and misuse of liothyronine in hypothyroidism. Nat Rev Endocrinol. (2024) 21:3–4. doi: 10.1038/s41574-024-01055-y, PMID: 39448832

[39] Hansen PS Brix TH Sorensen TI Kyvik KO Hegedüs L. Major genetic influence on the regulation of the pituitary-thyroid axis: a study of healthy Danish twins. J Clin Endocrinol Metab. (2004) 89:1181–7. doi: 10.1210/jc.2003-031641, PMID: 15001606

[40] De Grande LAC Goossens K Van Uytfanghe K Das B MacKenzie F Patru MM. Monitoring the stability of the standardization status of FT4 and TSH assays by use of daily outpatient medians and flagging frequencies. Clin Chim Acta. (2017) 467:8–14. doi: 10.1016/j.cca.2016.04.032, PMID: 27132242

[41] Razvi S Bhana S Mrabeti S. Challenges in interpreting thyroid stimulating hormone results in the diagnosis of thyroid dysfunction. J Thyroid Res. (2019) 2019:4106816. doi: 10.1155/2019/4106816, PMID: 31662841 PMC6778876

[42] Taylor PN Iqbal A Minassian C Sayers A Draman MS Greenwood R. Falling threshold for treatment of borderline elevated thyrotropin levels-balancing benefits and risks: evidence from a large community-based study. JAMA Intern Med. (2014) 174:32–9. doi: 10.1001/jamainternmed.2013.11312, PMID: 24100714

[43] Bengtsson E Funkquist A Agvall B. Observational study of diagnosis and management in adult primary hypothyroidism in southwest of Sweden. Scand J Prim Health Care. (2023) 24:1–7. doi: 10.1080/02813432.2023.2213748, PMID: 37224192 PMC10478599

[44] Berger C Calabrese R. Uncertainty Reduction. Oxford, UK: Communication Theory (2005).

[45] Mitchell AL Hegedüs L Zarkovic M Hickey JL Perros P. Patient satisfaction and quality of life in hypothyroidism: An online survey by the British Thyroid Foundation. Clin Endocrinol (Oxf). (2021) 94:513–20. doi: 10.1111/cen.14340, PMID: 32978985

[46] Enders A Uscinski J Seelig M Klofstad C Wuchty S Funchion J. The relationship between social media use and beliefs in conspiracy theories and misinformation. J Polit Behav. (2023) 45:781–804. doi: 10.1007/s11109-021-09734-6, PMID: 34248238 PMC8262430

[47] Perros P. Seeking thyroid truths: a guide for the curious. Cham: Springer (2024).

[48] Singh K Lima G Cha M Cha C Kulshrestha J Ahn YY. Misinformation, believability, and vaccine acceptance over 40 countries: takeaways from the initial phase of the COVID-19 infodemic. PloS One. (2022) 17:e0263381. doi: 10.1371/journal.pone.0263381, PMID: 35139117 PMC8827463

[49] Attanasio R Žarković M Papini E Nagy EV Negro R Perros P. Patients’ persistent symptoms, clinician demographics and geo-economic factors are associated with choice of therapy for hypothyroidism by European thyroid specialists: the “THESIS”* collaboration (*Treatment of Hypothyroidism in Europe by Specialists, an International Survey). Thyroid. (2024) 34:429–41. doi: 10.1089/thy.2023.0580, PMID: 38368541

[50] Žarković M Attanasio R Nagy EV Negro R Papini E Perros P. Characteristics of specialists treating hypothyroid patients: the “THESIS” Collaborative* *Treatment of hypothyroidism in europe by specialists: an international survey. Front Endocrinol. (2023) 14:1225202. doi: 10.3389/fendo.2023.1225202, PMID: 38027187 PMC10660282

[51] Papini E Attanasio R Žarković M Nagy E Negro R Perros P. Thyroid hormones for euthyroid patients with simple goiter growing over time: a survey of European thyroid specialists. Endocrine. (2025) 87:262–72. doi: 10.1007/s12020-024-04002-z, PMID: 39217207

[52] Negro R Žarković M Attanasio R Hegedüs L Nagy E Papini E. Use of levothyroxine for euthyroid, thyroid antibody positive women with infertility: Analyses of aggregate data from a survey of European thyroid specialists (Treatment of Hypothyroidism in Europe by Specialists: An International Survey). Clin Endocrinol (Oxf). (2024) 101:180–90. doi: 10.1111/cen.15099, PMID: 38856700

[53] Galofré CG Díez JJ Attanasio R Nagy EV Negro R Papini E. Treatment of obesity with thyroid hormones in Europe. Data from the THESIS* Collaboration. J Endocrinol Invest. (2024) 48:201–12. doi: 10.1007/s40618-024-02409-z, PMID: 38878126 PMC11729071

