# Supplementary material for: Knowledge gaps about the diagnosis and treatment of hypothyroidism: an international patient survey
Source: Front Endocrinol (Lausanne). 2025 Aug 29;16:1663497. doi: 10.3389/fendo.2025.1663497 (PMC12425718; doi:10.3389/fendo.2025.1663497)
Supplement: Supplementary file 1 [file DataSheet1.docx]

Supplementary Material

# Supplementary Data

**SUPPLEMENT 1**

**Questionnaire**

If A2. Are you 18 years of age or over? No Is Selected

If Are you currently taking anything to control symptoms of hypothyroidism?

Or Are you currently taking anything to control symptoms of hypothyroidism?

What is this questionnaire about? 
This questionnaire is about your care experiences and support needs as someone who is being treated for hypothyroidism (e.g. due to an under-active thyroid, not having a thyroid, or treatments for hyperthyroidism). You may have taken part in the pilot version of this questionnaire a few months ago. Please note that we would still value your input even if you participated in an earlier pilot questionnaire. Results from the survey will be used to better understand the different experiences of those with hypothyroidism. More information is available [here](https://www.picker.org/patient-experiences-of-hypothyroidism-care-and-treatment/).

This questionnaire is sponsored by the pharmaceutical company, IBSA, with the involvement of Thyroid Federation International and an academic board. The purpose is non-commercial and the data will be used for publication in the medical press. 

Who is this questionnaire for?   
This questionnaire is for adults aged 18 years and over who are being treated for hypothyroidism.   

If you have any questions about how to complete this questionnaire please email Harriet Hay at [take_part@pickereurope.ac.uk](mailto:take_part@pickereurope.ac.uk?subject=Hypothyroidism%20Patient%20Experience%20Survey) and reference 'Hypothyroidism Patient Experience Survey' within the subject line.   This survey will take approximately 30 minutes to complete. For ease of completion, we recommend completing this on a computer screen within one session.  The care you received may have been impacted by the Coronavirus pandemic. Please answer based on your typical experience or according to date ranges mentioned in the question text, excluding any instances where your care may be atypical due to the pandemic.    Taking part in this survey is voluntary. Your answers will be anonymised and treated in confidence.
You can access the Picker privacy notice for online surveys [here](https://www.picker.org/privacy-notice-for-online-surveys/).

R1 By completing this questionnaire, you are confirming that you are happy for Picker to use the anonymous data gathered. A summary of this information may be shared publicly for the benefit of others. Are you happy for your data to be shared?

- Yes, I am happy for my data to be shared anonymously (1)
- No, I do not want my data to be shared (2)

R0 If you close the questionnaire before the end of the survey, we would still like to use the responses that you give, even if you do not finish the survey. Are you happy for us to do so?

- Yes, you may use my responses if I only partly complete the survey (1)
- No, you may not use my responses unless I fully complete the survey (2)

A2. Are you 18 years of age or over?

- Yes (1)
- No (2)

A10 Are you currently taking anything to control symptoms of hypothyroidism (e.g. due to an under-active thyroid, not having a thyroid, or treatments for hyperthyroidism)? This may include prescribed medications, non-prescribed medications, and dietary supplements.

- Yes (1)
- No (2)
- Don't Know (99)

A3 What is your gender?

- Male (1)
- Female (2)
- Prefer to self-identify (3) ________________________________________________
- Prefer not to say (4)

A4 Where do you currently live?

D1 When did you develop hypothyroidism?

- Don't Know/ Can't remember (99)
- Within the last year (1)
- More than 1 year but less than 2 years ago (2)
- More than 2 years but less than 5 years ago (3)
- More than 5 years but less than 10 years ago (4)
- More than 10 years ago (5)

D2 What is the likely cause of your hypothyroidism?

- Not known (99)
- Hashimoto/autoimmune disease (1)
- Treatment for Graves' disease or hyperthyroidism (2)
- Treatment for thyroid cancer (3)
- Treatment for benign goiter (4)
- Medications (6)
- Pituitary disease (7)
- Congenital disorder (e.g. born without a thyroid or with a defective thyroid) (8)
- Pregnancy related (9)
- Wilson's temperature syndrome (11)
- Other (Please specify) (12) ________________________________________________

TSH stands for Thyroid Stimulating Hormone. A TSH test is a blood test that measures this hormone level.

D3a How long ago was your most recent TSH test?

- Don't know/ Can't remember (99)
- Within the last 2 months (1)
- Between 2-6 months ago (2)
- Between 6-12 months ago (3)
- More than 12 months ago (4)

D3b In what range was your current or most recently measured TSH test?

- Don't Know/ Can't remember (99)
- Under 0.1 (1)
- Between 0.1 and 0.4 (2)
- Over 0.4 but less than 2.5 (3)
- Between 2.5 and 4.0 (4)
- Between 4.0 and 10 (5)
- Between 10 and 20 (6)
- More than 20 (7)

D4 Thinking about your highest ever TSH level, in what range did it fall in?

- Don't Know/ Can't remember (99)
- Under 4.0 (4)
- Between 4.0 and 10 (5)
- Between 10 and 20 (6)
- More than 20 (7)

D5 What are you currently taking to treat your hypothyroidism? (Please select all that apply).

- Levothyroxine (e.g. Levoxyl, Synthroid, Euthyrox, Unithroid, Eltroxin, Levothyroid, Tyrosint, L Thyroxine, Levo T, Levoxine, Levothyroxine, T4) (1)
- Liothyronine (e.g. T3, Cytomel, Triostat, Tertroxin, Thybon) (2)
- Desiccated Thyroid Extract (e.g. Armour Thyroid, Nature-Throid, NP Thyroid, ERFA Thyroid, Westhroid) (3)
- Dietary supplements (4)
- Other treatment (Please specify) (5)
- ⊗Don't know/Prefer not to say (99)

D6 Have you ever taken Levothyroxine (e.g. Levoxyl, Synthroid, Euthyrox, Unithroid, Eltroxin, Levothyroid, Tyrosint, L Thyroxine, Levo T, Levoxine, Levothyroxine, T4) to treat your hypothyroidism?

- Yes (1)
- No (2)
- Don't know/can't remember (99)

D7 How long did you take Levothyroxine (e.g. Levoxyl, Synthroid, Euthyrox, Unithroid, Eltroxin, Levothyroid, Tyrosint, L Thyroxine, Levo T, Levoxine, Levothyroxine, T4)?

- Less than 1 year (1)
- More than 1 year but less than 2 years (2)
- More than 2 years but less than 5 years (3)
- More than 5 years but less than 10 years (4)
- 10 years or more (5)
- Don't know/can't remember (99)
- D8 How long have you been taking Levothyroxine (e.g. Levoxyl, Synthroid, Euthyrox, Unithroid, Eltroxin, Levothyroid, Tyrosint, L Thyroxine, Levo T, Levoxine, Levothyroxine, T4)?
- Less than 1 year (1)
- More than 1 year but less than 2 years (2)
- More than 2 years but less than 5 years (3)
- More than 5 years but less than 10 years (4)
- 10 years or more (5)
- Don't know/can't remember (99)

D9 What is the primary reason why you stopped taking Levothyroxine (e.g.Levoxyl, Synthroid, Euthyrox, Unithroid, Eltroxin, Levothyroid, Tyrosint, L Thyroxine, Levo T, Levoxine, Levothyroxine T4)? (Please select all that apply).

- I wanted to take something more natural (1)
- I needed to change treatments due to a history of thyroid cancer (2)
- Levothyroxine wasn't controlling my symptoms well (3)
- Levothyroxine was interacting with other medications I take (4)
- I was experiencing unpleasant side effects (5)
- To avoid an ingredient in the medication (e.g. gelatine, lactose) (6)
- I was advised by my doctor to do so (7)
- Other (please specify) (8)
- ⊗Don't know/can't remember (99)

M1 In what form do you currently take your Levothyroxine (e.g. Levoxyl, Synthroid, Euthyrox, Unithroid, Eltroxin, Levothyroid, Tyrosint, L Thyroxine, Levo T, Levoxine, Levothyroxine, T4)?

- Tablets (1)
- Capsules (2)
- Soft gels (3)
- Liquid (5)
- Don't Know (99)

M2 How many times do you take your Levothyroxine (e.g. Levoxyl, Synthroid, Euthyrox, Unithroid, Eltroxin, Levothyroid, Tyrosint, L Thyroxine, Levo T, Levoxine, Levothyroxine, T4)?

- Once a day (1)
- Twice a day (2)
- Three times per day (3)
- More than three times per day (4)
- Once a week (5)
- Other (Please specify) (6) ________________________________________________
- Don't know (99)

M28 How many times do you take your Liothyronine (e.g. Cytomel, Triostat, Tertroxin, Thybon)?

- Once a day (1)
- Twice a day (2)
- Three times per day (3)
- More than three times per day (4)
- Other (Please specify) (5) ________________________________________________

M29 How many times do you take your Desiccated Thyroid Extract (e.g. Armour Thyroid, Nature-Throid, NP Thyroid, ERFA Thyroid, Westhroid)?

- Once a day (1)
- Twice a day (2)
- Three times per day (3)
- More than three times per day (4)
- Other (Please specify) (5)

M25 Do you currently take alternate doses of your Levothyroxine medication (e.g. Levoxyl, Synthroid, Euthyrox, Unithroid, Eltroxin, Levothyroid, Tyrosint, L Thyroxine, Levo T, Levoxine, Levothyroxine, T4)? For example, you might take 100 micrograms (mcg) on one day and 75 micrograms (mcg) the next day.

- Yes (1)
- No (2)
- Don't Know/can't remember (99)

M26 What is the total dose you are supposed to take each day you have your Levothyroxine (e.g. Levoxyl, Synthroid, Euthyrox, Unithroid, Eltroxin, Levothyroid, Tyrosint, L Thyroxine, Levo T, Levoxine, Levothyroxine, T4)?N(If you only take your dose once a week, please provide the weekly dosage).

- ⊗Dose (enter number below in micrograms) (1)
- Don't know/can't remember (99)

M27 You said that you take alternate doses when you have your Levothyroxine.
What are the two doses you are supposed to take each time you have your Levothyroxine (e.g. Levoxyl, Synthroid, Euthyrox, Unithroid, Eltroxin, Levothyroid, Tyrosint, L Thyroxine, Levo T, Levoxine, Levothyroxine, T4)?

- First dose (enter number below in micrograms) (1)
- Second dose (enter number below in micrograms) (2)
- ⊗Don't Know/can't remember (99)

M14 What is the total dose you are supposed to take each day you have your Liothyronine (e.g. Cytomel, Triostat, Tertroxin, Thybon)?

- ⊗Less than 5 micrograms (1)
- ⊗5-20 micrograms (99)
- 21-60 micrograms (100)
- 61-120 micrograms (101)
- More than 121 micrograms (102)

M15 What is the total number of grains/tablets you are supposed to take each day you have your Dessicated Thyroid Extract (e.g. Armour Thyroid, Nature-Throid, NP Thyroid, ERFA Thyroid, Westhroid)?

- Don't Know/can't remember (99)
- ⊗Less than 1 grain/tablet (1)
- 1 -2 grains/tablets (100)
- 2.25 - 3 grains/tablets (101)
- 3.25 grains/tablets or more (102)

M17 In the past 12 months, how many times has the dose of your hypothyroidism medication been adjusted?

- Never (1)
- Once (2)
- Twice (3)
- Three times (4)
- Four times (5)
- Five times or more (6)
- Don't know/can't remember (99)

M18 Thinking about taking your hypothyroidism medication in the past 12 months, how burdensome (if at all) have any of the following been? 
  Please select one option from each row. Select ‘This does not apply to me’ if you do not need to follow the guideline. Some statements may or may not apply to you. Select ‘Not burdensome at all’ if you do need to follow the guideline but do not find it burdensome.

|  | This does not apply to me (98) | Not burdensome at all (1) | Slightly burdensome (2) | Moderately burdensome (3) | Extremely burdensome (4) |
| --- | --- | --- | --- | --- | --- |
| Making sure that I take the correct dose at the right time (M18a) |  |  |  |  |  |
| Making sure that I take the dose on an empty stomach (M18b) |  |  |  |  |  |
| Making sure that I avoid drinking anything other than water for 30 minutes before and after taking my medication (M18c) |  |  |  |  |  |
| Making sure that I do not take any other medications that could interfere with my hypothyroidism medication (M18d) |  |  |  |  |  |
| *M25 = Yes*  Needing to alternate doses in order to get the right amount of levothyroxine (M18e) |  |  |  |  |  |
| Needing to split tablets in order to get the right dose (M18f) |  |  |  |  |  |

M19 Still thinking about taking your hypothyroidism medication in the past 12 months, how burdensome (if at all) have any of the following been?    Please select one option from each row. Select ‘This does not apply to me’ if you do not need to follow the guideline. Some statements may or may not apply to you. Select ‘Not burdensome at all’ if you do need to follow the guideline but do not find it burdensome.

|  | This does not apply to me (98) | Not burdensome at all (1) | Slightly burdensome (2) | Moderately burdensome (3) | Extremely burdensome (4) |
| --- | --- | --- | --- | --- | --- |
| *If What are you currently taking to treat your hypothyroidism? (Please select all that apply). q://QID12/SelectedChoicesCount Is Greater Than 1*  Keeping track of my different hypothyroidism medications (M19a) |  |  |  |  |  |
| Having difficulty taking my medication because it tastes bad/does not look appealing (M19b) |  |  |  |  |  |
| Experiencing problems getting prescriptions from my doctor (M19c) |  |  |  |  |  |
| Making sure that I am getting blood tests done to make sure I am taking the right dose (M19d) |  |  |  |  |  |
| *M17 != Never*  Getting used to taking a different dose of my medication (M19e) |  |  |  |  |  |

M20 How often do you typically miss or skip taking a dose of your hypothyroidism medication?

- Most days (1)
- A few times per week (2)
- Once a week (3)
- A few times per month (4)
- Once a month (5)
- A few times per year (6)
- Never (7)

M21 Why have you missed or skipped taking your medication? (Please select all that apply).

- I forgot (1)
- My routine was disrupted / I did not have the medication with me (2)
- I could not meet the food/drink requirements (3)
- I experienced unpleasant side effects (4)
- I wanted to avoid an ingredient in the medication (e.g. gelatine, lactose) (5)
- I didn’t have any symptoms (6)
- I was afraid the medication would interact with other medication I had to take (7)
- I didn’t think that the medication was working (8)
- I was feeling too ill to take it (9) *A3 = Female*
- I had concerns due to pregnancy (10)
- I ran out of medication (11)
- I needed additional information or support to take my medication (12)
- I could not afford the medication (13)
- Other (please specify) (14)

M22 How often do you typically take more of your hypothyroidism medication than what you have been prescribed?

- Most days (1)
- A few times per week (2)
- Once a week (3)
- A few times per month (4)
- Once a month (5)
- A few times per year (6)
- Never (7)

M23 Why have you taken more of your hypothyroidism medication than what you have been prescribed? (Please select all that apply)

- I missed a dose and had to take more later (1)
- I forgot that I had already taken a dose and took it again (2)
- I felt like I needed to take more to control my symptoms (3)
- I had difficulty knowing how much to take (4)
- Other (please specify) (5) *Display This Question:*

M24 To what extent, if at all, do you agree or disagree with the following statements? (Please select one option from each row).

|  | Strongly agree (1) | Tend to agree (2) | Neither agree nor disagree (3) | Tend to disagree (4) | Strongly disagree (5) |
| --- | --- | --- | --- | --- | --- |
| My hypothyroidism medication controls my symptoms well (M24a) |  |  |  |  |  |
| My hypothyroidism medication is convenient to take (M24b) |  |  |  |  |  |

Treatment Expectations

T1 When you first started taking levothyroxine for hypothyroidism, did you expect that your overall health would…

- Improve significantly (1)
- Improve slightly (2)
- Be about the same (3)
- I did not have any expectations (4)
- Don’t know/can’t remember (99)

T2 After you started taking levothyroxine for hypothyroidism, when did you expect the treatment to start working?

- Immediately (1)
- Within 1 - 3 months (2)
- Within 4 - 12 months (3)
- Within 13 months - 2 years (4)
- I did not have any expectations (5)
- Don’t know/can’t remember (99)

T3 How concerned were you about weight gain before starting treatment for hypothyroidism?

- Very concerned (1)
- Moderately concerned (2)
- A little concerned (3)
- Not at all concerned (4)
- Don’t know/can’t remember (99)

T4 How concerned are you about weight gain now?

- Very concerned (1)
- Moderately concerned (2)
- A little concerned (3)
- Not at all concerned (4)

T5A Thinking about your treatment for hypothyroidism, how did the introduction of ${D5/ChoiceDescription/2} make you feel?

- Very satisfied compared to before (1)
- A little more satisfied than before (2)
- Neither satisfied nor dissatisfied (3)
- A little less satisfied than before (4)
- Very dissatisfied compared to before (5)
- Don’t know (99)

T5B Thinking about your treatment for hypothyroidism, how did the introduction of ${D5/ChoiceDescription/2} or ${D5/ChoiceDescription/3} make you feel?

- Very satisfied compared to before (1)
- A little more satisfied than before (2)
- Neither satisfied nor dissatisfied (3)
- A little less satisfied than before (4)
- Very dissatisfied compared to before (5)
- Don’t know (99)

T5C Thinking about your treatment for hypothyroidism, how did the introduction of ${D5/ChoiceDescription/3} make you feel?

- Very satisfied compared to before (1)
- A little more satisfied than before (2)
- Neither satisfied nor dissatisfied (3)
- A little less satisfied than before (4)
- Very dissatisfied compared to before (5)
- Don’t know (99)

Description Other conditions and medications

C1 Do you have a current diagnosis of any of the following conditions? (Please select all that apply).

- ⊗No long-term condition (17)
- Autoimmune disease (other than thyroid) (1)
- Heart disease (e.g. angina, coronary artery disease, congenital heart disease) (2)
- Lung disease (e.g. asthma, COPD) (3)
- Diabetes (4)
- Joint problem (e.g. arthritis or other rheumatic disease) (5)
- Osteoporosis (6)
- Bone/muscle disease (e.g. fibromyalgia) (7)
- Gastrointestinal (GI) disease (e.g. irritable bowel syndrome, celiac disease) (8)
- Mental health condition (e.g. depression, anxiety) (9)
- Cancer (10)
- Chronic pain (e.g. back pain) (11)
- Fatigue syndrome (e.g. chronic fatigue, myalgic encephalomyelitis) (12)
- Adrenal disease (e.g. adrenal fatigue, Addison’s disease) (13)
- Food allergy (e.g. gluten intolerance) (14)
- Sleep apnoea (15)
- Other long-term condition (16)

C2 Do you take prescribed medications for any condition other than hypothyroidism?

- Yes (1)
- No (2)

C3 In addition to hypothyroidism, how many conditions do you take prescribed medicine for?

H1 In a typical week, how many hours do you spend doing physical activity, which is enough to raise your breathing rate? (This may include sport, exercise, and brisk walking or cycling for recreation or to get to and from places, but should NOT include housework or physical activity that may be part of your job).

- None (1)
- Less than 1 hour a week (2)
- 1 to 3 hours a week (3)
- 3 to 6 hours a week (4)
- 6 to 10 hours a week (5)
- 10 to 15 hours a week (6)
- More than 15 hours a week (7)

H2 Given your age and height, would you say that you are about the right weight, too heavy, or too light?

- I am about the right weight (1)
- I am too heavy (2)
- I am too light (3)
- Don't know (99)

H3 During the past 4 weeks, how much have you been bothered by any of the following problems?

|  | Not bothered at all/Not applicable (0) | Bothered a little (1) | Bothered a lot (2) |
| --- | --- | --- | --- |
| Stomach pain (H3a) |  |  |  |
| Memory problems (H3b) |  |  |  |
| Weight loss (H3c) |  |  |  |
| *A3 != Male*  Menstrual cramps or other problems with your periods (H3d) |  |  |  |
| Sensitivity to the cold (H3e) |  |  |  |
| Shaking, usually of the hands (H3f) |  |  |  |
| Headaches (H3g) |  |  |  |
| Dry/itchy skin (H3h) |  |  |  |
| Flushing or sweating a lot (H3i) |  |  |  |
| Chest pain (H3j) |  |  |  |
| Pins and needles in the fingers and hands (H3k) |  |  |  |
| Mood swings (H3l) |  |  |  |
| Feeling your heart pound or race (H3m) |  |  |  |

H4 You mentioned that you were bothered by the following problems during the past 4 weeks. What do you think is the main cause or causes of each of these problems?

|  | My hypothyroidism or side effects from my hypothyroidism medication (1) | Another condition or its medication (2) | Ageing (3) | Lifestyle (e.g. diet, exercise, work) (4) | Short term illness (e.g. cold/ flu/ allergies) (5) | Other (6) | Don't know (99) |
| --- | --- | --- | --- | --- | --- | --- | --- |
| *H3 = Stomach pain [ Bothered a little ]*  *Or H3 = Stomach pain [ Bothered a lot ]*  Stomach pain (H4a) |  |  |  |  |  |  |  |
| *H3 = Memory problems [ Bothered a little ]*  *Or H3 = Memory problems [ Bothered a lot ]*  Memory problems (H4b) |  |  |  |  |  |  |  |
| *H3 = Weight loss [ Bothered a little ]*  *Or H3 = Weight loss [ Bothered a lot ]*  Weight loss (H4c) |  |  |  |  |  |  |  |
| *H3 = Menstrual cramps or other problems with your periods [ Bothered a little ]*  *Or H3 = Menstrual cramps or other problems with your periods [ Bothered a lot ]*  Menstrual cramps or other problems with your periods (H4d) |  |  |  |  |  |  |  |
| *H3 = Sensitivity to the cold [ Bothered a little ]*  *Or H3 = Sensitivity to the cold [ Bothered a lot ]*  Sensitivity to the cold (H4e) |  |  |  |  |  |  |  |
| *H3 = Shaking, usually of the hands [ Bothered a little ]*  *Or H3 = Shaking, usually of the hands [ Bothered a lot ]*  Shaking, usually of the hands (H4f) |  |  |  |  |  |  |  |
| *H3 = Headaches [ Bothered a little ]*  *Or H3 = Headaches [ Bothered a lot ]*  Headaches (H4g) |  |  |  |  |  |  |  |
| *H3 = Dry/itchy skin [ Bothered a little ]*  *Or H3 = Dry/itchy skin [ Bothered a lot ]*  Dry/itchy skin (H4h) |  |  |  |  |  |  |  |
| *H3 = Flushing or sweating a lot [ Bothered a little ]*  *Or H3 = Flushing or sweating a lot [ Bothered a lot ]*  Flushing or sweating a lot (H4i) |  |  |  |  |  |  |  |
| *H3 = Chest pain [ Bothered a little ]*  *Or H3 = Chest pain [ Bothered a lot ]*  Chest pain (H4j) |  |  |  |  |  |  |  |
| *H3 = Pins and needles in the fingers and hands [ Bothered a little ]*  *Or H3 = Pins and needles in the fingers and hands [ Bothered a lot ]*  Pins and needles in the fingers and hands (H4k) |  |  |  |  |  |  |  |
| *H3 = Mood swings [ Bothered a little ]*  *Or H3 = Mood swings [ Bothered a lot ]*  Mood swings (H4l) |  |  |  |  |  |  |  |
| *H3 = Feeling your heart pound or race [ Bothered a little ]*  *Or H3 = Feeling your heart pound or race [ Bothered a lot ]*  Feeling your heart pound or race (H4m) |  |  |  |  |  |  |  |

H5 During the past 4 weeks, how much have you been bothered by any of the following problems?

|  | Not bothered at all/Not applicable (0) | Bothered a little (1) | Bothered a lot (2) |
| --- | --- | --- | --- |
| Weight gain (H5n) |  |  |  |
| Irritability (H5o) |  |  |  |
| Constipation, loose bowels, or diarrhoea (H5p) |  |  |  |
| Slow speech, movements, or thoughts (H5q) |  |  |  |
| Feeling tired or having low energy (H5r) |  |  |  |
| Low mood or depression (H5s) |  |  |  |
| Anxiety (H5t) |  |  |  |
| Trouble sleeping (H5u) |  |  |  |
| Difficulty concentrating (H5v) |  |  |  |
| Nervousness (H5w) |  |  |  |
| Back pain (H5x) |  |  |  |
| Dry hair/nails (H5y) |  |  |  |
| Feeling restless (H5z) |  |  |  |

H6 You mentioned that you were bothered by the following problems during the past 4 weeks. What do you think is the main cause or causes of each of these problems?

|  | My hypothyroidism or side effects from my hypothyroidism medication (1) | Another condition or its medication (2) | Ageing (3) | Lifestyle (e.g. diet, exercise, work) (4) | Short term illness (e.g. cold/ flu/ allergies) (5) | Other (6) | Don't know (99) |
| --- | --- | --- | --- | --- | --- | --- | --- |
| *H5 = Weight gain [ Bothered a little ]*  *Or H5 = Weight gain [ Bothered a lot ]*  Weight gain (H6n) |  |  |  |  |  |  |  |
| *H5 = Irritability [ Bothered a little ]*  *Or H5 = Irritability [ Bothered a lot ]*  Irritability (H6o) |  |  |  |  |  |  |  |
| *H5 = Constipation, loose bowels, or diarrhoea [ Bothered a little ]*  *Or H5 = Constipation, loose bowels, or diarrhoea [ Bothered a lot ]*  Constipation, loose bowels, or diarrhea (H6p) |  |  |  |  |  |  |  |
| *H5 = Slow speech, movements, or thoughts [ Bothered a little ]*  *Or H5 = Slow speech, movements, or thoughts [ Bothered a lot ]*  Slow speech, movements, or thoughts (H6q) |  |  |  |  |  |  |  |
| *H5 = Feeling tired or having low energy [ Bothered a little ]*  *Or H5 = Feeling tired or having low energy [ Bothered a lot ]*  Feeling tired or having low energy (H6r) |  |  |  |  |  |  |  |
| *H5 = Low mood or depression [ Bothered a little ]*  *Or H5 = Low mood or depression [ Bothered a lot ]*  Low mood or depression (H6s) |  |  |  |  |  |  |  |
| *H5 = Anxiety [ Bothered a little ]*  *Or H5 = Anxiety [ Bothered a lot ]*  Anxiety (H6t) |  |  |  |  |  |  |  |
| *H5 = Trouble sleeping [ Bothered a little ]*  *Or H5 = Trouble sleeping [ Bothered a lot ]*  Trouble sleeping (H6u) |  |  |  |  |  |  |  |
| *H5 = Difficulty concentrating [ Bothered a little ]*  *Or H5 = Difficulty concentrating [ Bothered a lot ]*  Difficulty concentrating (H6v) |  |  |  |  |  |  |  |
| *H5 = Nervousness [ Bothered a little ]*  *Or H5 = Nervousness [ Bothered a lot ]*  Nervousness (H6w) |  |  |  |  |  |  |  |
| *H5 = Back pain [ Bothered a little ]*  *Or H5 = Back pain [ Bothered a lot ]*  Back pain (H6x) |  |  |  |  |  |  |  |
| *H5 = Dry hair/nails [ Bothered a little ]*  *Or H5 = Dry hair/nails [ Bothered a lot ]*  Dry hair/nails (H6y) |  |  |  |  |  |  |  |
| *H5 = Feeling restless [ Bothered a little ]*  *Or H5 = Feeling restless [ Bothered a lot ]*  Feeling restless (H6z) |  |  |  |  |  |  |  |

H7 During the past 4 weeks, how much have you been bothered by any of the following problems?

|  | Not bothered at all/Not applicable (0) | Bothered a little (1) | Bothered a lot (2) |
| --- | --- | --- | --- |
| Pain in your arms, legs, or joints (knees, hips, etc.) (H7a) |  |  |  |
| Hoarse/croaky voice (H7b) |  |  |  |
| Thinning hair (H7c) |  |  |  |
| Dizziness (H7d) |  |  |  |
| Puffy face/bags under eyes (H7e) |  |  |  |
| Low sex drive (H7f) |  |  |  |
| Fainting spells (H7g) |  |  |  |
| Hearing loss (H7h) |  |  |  |
| Muscle weakness/cramps/aches (H7i) |  |  |  |
| Shortness of breath (H7j) |  |  |  |
| Nausea, gas, or indigestion (H7k) |  |  |  |
| Pain or problems during sexual intercourse (H7l) |  |  |  |

H8 You mentioned that you were bothered by the following problems during the past 4 weeks. What do you think is the main cause or causes of each of these problems?

|  | My hypothyroidism or side effects from my hypothyroidism medication (1) | Another condition or its medication (2) | Ageing (3) | Lifestyle (e.g. diet, exercise, work) (4) | Short term illness (e.g. cold/ flu/ allergies) (5) | Other (6) | Don't know (99) |
| --- | --- | --- | --- | --- | --- | --- | --- |
| *H7 = Pain in your arms, legs, or joints (knees, hips, etc.) [ Bothered a little ]*  *Or H7 = Pain in your arms, legs, or joints (knees, hips, etc.) [ Bothered a lot ]*  Pain in your arms, legs, or joints (knees, hips, etc.) (H8a) |  |  |  |  |  |  |  |
| *H7 = Hoarse/croaky voice [ Bothered a little ]*  *Or H7 = Hoarse/croaky voice [ Bothered a lot ]*  Hoarse/croaky voice (H8b) |  |  |  |  |  |  |  |
| *H7 = Thinning hair [ Bothered a little ]*  *Or H7 = Thinning hair [ Bothered a lot ]*  Thinning hair (H8c) |  |  |  |  |  |  |  |
| *H7 = Dizziness [ Bothered a little ]*  *Or H7 = Dizziness [ Bothered a lot ]*  Dizziness (H8d) |  |  |  |  |  |  |  |
| *H7 = Puffy face/bags under eyes [ Bothered a little ]*  *Or H7 = Puffy face/bags under eyes [ Bothered a lot ]*  Puffy face/bags under eyes (H8e) |  |  |  |  |  |  |  |
| *H7 = Low sex drive [ Bothered a little ]*  *Or H7 = Low sex drive [ Bothered a lot ]*  Low sex drive (H8f) |  |  |  |  |  |  |  |
| *H7 = Fainting spells [ Bothered a little ]*  *Or H7 = Fainting spells [ Bothered a lot ]*  Fainting spells (H8g) |  |  |  |  |  |  |  |
| *H7 = Hearing loss [ Bothered a little ]*  *Or H7 = Hearing loss [ Bothered a lot ]*  Hearing loss (H8h) |  |  |  |  |  |  |  |
| *H7 = Muscle weakness/cramps/aches [ Bothered a little ]*  *Or H7 = Muscle weakness/cramps/aches [ Bothered a lot ]*  Muscle weakness/cramps/aches (H8i) |  |  |  |  |  |  |  |
| *H7 = Shortness of breath [ Bothered a little ]*  *Or H7 = Shortness of breath [ Bothered a lot ]*  Shortness of breath (H8j) |  |  |  |  |  |  |  |
| *H7 = Nausea, gas, or indigestion [ Bothered a little ]*  *Or H7 = Nausea, gas, or indigestion [ Bothered a lot ]*  Nausea, gas, or indigestion (H8k) |  |  |  |  |  |  |  |
| *H7 = Pain or problems during sexual intercourse [ Bothered a little ]*  *Or H7 = Pain or problems during sexual intercourse [ Bothered a lot ]*  Pain or problems during sexual intercourse (H8l) |  |  |  |  |  |  |  |

S5 Have you been seen by healthcare staff in the past 12 months about your hypothyroidism?

- Yes (1)
- No (2)

Thinking about the care you have received for your hypothyroidism over the past 12 months from the healthcare staff who you primarily see for your thyroid...

S1 Do you have confidence and trust in the healthcare staff treating your hypothyroidism?

- Yes, always (1)
- Yes, sometimes (2)
- No (3)

S2 Do the healthcare staff that you see for your hypothyroidism know enough about the condition?

- Yes, definitely (1)
- Yes, to some extent (2)
- No (3)

S3 Do you have enough time to talk and interact with healthcare staff about your hypothyroidism?

- Yes, definitely (1)
- Yes, to some extent (2)
- No (3)

S4 Do healthcare staff talk to you about your care and treatment in a way that you can understand?

- Yes, definitely (1)
- Yes, to some extent (2)
- No (3)

Thinking about managing your condition over the past 12 months...

SM1 Have you been given enough information about when and how to take your hypothyroidism medication?

- Yes, enough information (1)
- Some, but not enough information (2)
- Little or no information (3)
- I did not need any information (4)

SM2 Have you been given enough information about the side effects of your hypothyroidism medication and any interactions with other medications and supplements?

- Yes, enough information (1)
- Some, but not enough information (2)
- Little or no information (3)
- I did not need any information (4)

SM3 Are you involved enough in decisions about your condition and treatment?

- Yes, definitely (1)
- Yes, to some extent (2)
- No, but I would like this (3)
- I do not want or need to be (4)

O1 How satisfied are you with the overall care and treatment you have received for your hypothyroidism?

- Very satisfied (1)
- Slightly satisfied (2)
- Neither satisfied nor dissatisfied (3)
- Slightly dissatisfied (4)
- Very dissatisfied (5)
- Don’t know (99)

*The care you received may have been impacted by the Coronavirus pandemic. Please answer based on your typical experience or according to date ranges mentioned in the question text, excluding any instances where your care may be atypical due to the pandemic.*
Thinking about how your hypothyroidism has affected your day-to-day life in the past 12 months...

Q1 To what extent, if at all, do you agree or disagree with each of the following statements? (Please select one option from each row).

|  | Strongly agree (1) | Tend to agree (2) | Neither agree nor disagree (3) | Tend to disagree (4) | Strongly disagree (5) | Don't know/can't recall (99) | This does not apply to me (98) |
| --- | --- | --- | --- | --- | --- | --- | --- |
| My hypothyroidism has affected everyday activities that people my age usually do (e.g. exercise, household chores, etc.) (Q1a) |  |  |  |  |  |  |  |
| Managing treatment or medications for my hypothyroidism has had a significant impact on my day-to-day life (Q1b) |  |  |  |  |  |  |  |
| My hypothyroidism has negatively impacted on my holiday/ vacation/travel plans (Q1c) |  |  |  |  |  |  |  |
| I have been unable to work/had to change my job or working pattern because of my hypothyroidism (Q1d) |  |  |  |  |  |  |  |
| My hypothyroidism has had a negative impact on my financial situation (Q1e) |  |  |  |  |  |  |  |

Q2 Still thinking about the past 12 months... 
To what extent, if at all, do you agree or disagree with each of the following statements? (Please select one option from each row)

|  | Strongly agree (1) | Tend to agree (2) | Neither agree nor disagree (3) | Tend to disagree (4) | Strongly disagree (5) | Don't know/can't recall (99) | This does not apply to me (98) |
| --- | --- | --- | --- | --- | --- | --- | --- |
| My hypothyroidism has created problems with my partner, close friends or relatives (Q2a) |  |  |  |  |  |  |  |
| My hypothyroidism has had a negative impact on my social life (Q2b) |  |  |  |  |  |  |  |
| My hypothyroidism has negatively impacted on my confidence and self-esteem (Q2c) |  |  |  |  |  |  |  |

We are interested in exploring whether there is any relationship between hypothyroidism and certain personality traits. Below are a number of statements that people often use to describe themselves. Please read each statement and then choose the most appropriate answer. There are no right or wrong answers: Your own impression is the only thing that matters. *Some statements may be affected by social distancing measures due to the Coronavirus pandemic. Please answer based on your typical experience, not based on measures you may have needed to take due to the pandemic.*

P1 I make contact easily when I meet people

- False (0)
- Rather false (1)
- Neutral (2)
- Rather true (3)
- True (4)

P2 I often make a fuss about unimportant things

- False (0)
- Rather false (1)
- Neutral (2)
- Rather true (3)
- True (4)

P3 I often talk to strangers

- False (0)
- Rather false (1)
- Neutral (2)
- Rather true (3)
- True (4)

P4 I often feel unhappy

- False (0)
- Rather false (1)
- Neutral (2)
- Rather true (3)
- True (4)

P5 I am often irritated

- False (0)
- Rather false (1)
- Neutral (2)
- Rather true (3)
- True (4)

P6 I often feel inhibited in social interactions

- False (0)
- Rather false (1)
- Neutral (2)
- Rather true (3)
- True (4)

P7 I take a gloomy view of things

- False (0)
- Rather false (1)
- Neutral (2)
- Rather true (3)
- True (4)

P8 I find it hard to start a conversation

- False (0)
- Rather false (1)
- Neutral (2)
- Rather true (3)
- True (4)

P9 I am often in a bad mood

- False (0)
- Rather false (1)
- Neutral (2)
- Rather true (3)
- True (4)

P10 I am a closed kind of person

- False (0)
- Rather false (1)
- Neutral (2)
- Rather true (3)
- True (4)

P11 I would rather keep other people at a distance

- False (0)
- Rather false (1)
- Neutral (2)
- Rather true (3)
- True (4)

P12 I often find myself worrying about something

- False (0)
- Rather false (1)
- Neutral (2)
- Rather true (3)
- True (4)

P13 I am often down in the dumps

- False (0)
- Rather false (1)
- Neutral (2)
- Rather true (3)
- True (4)

P14 When socializing, I don't find the right things to talk about

- False (0)
- Rather false (1)
- Neutral (2)
- Rather true (3)
- True (4)

Q135 We are interested in exploring your views on topics that often appear within patient forums.
*Some statements may be affected by social distancing measures due to the Coronavirus pandemic. Please answer based on your typical experience, not based on measures you may have needed to take due to the pandemic.* Please indicate whether you think the following statements are true or false. Please answer based on your own opinion. Note that the following statements ARE NOT medical statements. You should always follow the advice of your doctor. Experts have varying opinions about the statements that follow. We would like to hear yours.

|  | True (1) | False (2) | Don't know (99) |
| --- | --- | --- | --- |
| Untreated hypothyroidism can cause daily fluctuations of symptoms (I1a) |  |  |  |
| A patient with a normal thyroid blood test does not need to be treated with thyroid hormones (even if they have positive thyroid antibodies and symptoms) (I1b) |  |  |  |
| It's safe to be slightly over-treated with thyroid hormones (e.g. having a TSH below the normal range) (I1c) |  |  |  |
| Body temperature is the best method for diagnosing hypothyroidism (I1d) |  |  |  |
| Most patients with untreated hypothyroidism also have problems with their adrenal glands (I1e) |  |  |  |

I2 Please indicate whether you think the following statements are true or false. Please answer based on your own opinion. Note that the following statements ARE NOT medical statements. You should always follow the advice of your doctor. Experts have varying opinions about the statements that follow. We would like to hear yours.

|  | True (1) | False (2) | Don't know (99) |
| --- | --- | --- | --- |
| Hypothyroid patients need to take iodine supplements even if they are already taking thyroid hormone (I2a) |  |  |  |
| Having untreated hypothyroidism weakens the immune system and makes people prone to infection (I2b) |  |  |  |
| Hypothyroid patients can lose weight if adequately treated (I2c) |  |  |  |
| You can manage your hypothyroidism without medication, just by watching what you eat (I2d) |  |  |  |
| Hypothyroidism is an infectious disease (I2e) |  |  |  |

I3 To what extent do you use social media and the internet to find out information about your hypothyroidism?

- Daily (1)
- Once or twice a week (2)
- Once a month (3)
- Less than once a month (5)
- Never (4)

Q123 How old are you?

- 18-30 years (3)
- 31-40 years (9)
- 41-50 years (4)
- 51-60 years (5)
- 61-70 years (6)
- 71-80 years (7)
- 81 years or over (8)

A5 Please indicate your household status.

- Married/in a civil partnership/living with a partner (1)
- Single/divorced/widowed and living alone (2)
- Single/divorced/widowed and living with others (e.g. my parents, my children, and/or other adults) (3)
- Other (Please specify) (4) ________________________________________________
- Prefer not to say (5)

A6 Which statement best describes your employment status? If you are retired, disabled, or a student and are also working, then please choose the option 'Working (full time or part time)'.

- Working (full time, part time) (1)
- On maternity/parental leave (2)
- Not working - looking for work (3)
- Not working - retired (4)
- Not working - disabled/ on long-term sickness (5)
- Not working - Other (8)
- Full time carer (10)
- Student (6)
- Prefer not to say (9)
- Other (Please specify (11) ________________________________________________

A7 Which of these best describes your ethnic background?

- White (1)
- Mixed/multiple ethnic groups (2)
- Asian (3)
- Black/African/Afro-Caribbean (4)
- Middle Eastern/Arab (5)
- Latino (descended from Latin America) (6)
- Native American/Pacific Islander (7)
- Other (Please specify) (8) ________________________________________________
- Prefer not to say (9)

A8 How many years of education have you obtained? (Please include all education from primary/elementary through any secondary, vocational, university, and post-graduate education).

- Under 4 years (1)
- 4-8 years (2)
- 9-12 years (3)
- 13-16 years (4)
- 17-20 years (5)
- Over 20 years (6)
- Prefer not to say (7)

A9 How would you rate your household economic status (e.g. income, living conditions) compared to your country's average?

- Well above average (1)
- Above average (2)
- Average (3)
- Below average (4)
- Well below average (5)
- Don't know (6)
- Prefer not to say (7)

**SUPPLEMENT 2**

Independent variables used in the study. Where necessary, we recoded the survey data into a smaller number of categories to meet the assumptions of the chi square tests; for instance classifying “well above average” and “above average” to “above average” for the household income variable. With regards to anxiety and low mood / depression, participants were asked “during the past 4 weeks, how much have you been bothered by anxiety?” and “during the past 4 weeks, how much have you been bothered by low mood / depression?” with the following response options: “bothered a little” or “bothered a lot” (considered as having anxiety or low mood / depression), “not bothered at all” (considered as not having anxiety or low mood / depression).

Demographics

Gender

Age

Marital status

Employment status

Ethnic background

Countries

Years in education

Household income

Clinical variables

Number of comorbidities

Duration of hypothyroidism

Cause of hypothyroidism

Most recent serum TSH concentration

Highest ever recorded serum TSH

Treatment for hypothyroidism

Probable Somatic Symptom Disorder

Type D personality

Anxiety

Low mood/depression

Use of internet and social media to find information about hypothyroidism

Patient reported outcomes

Symptom control by treatment for hypothyroidism; participants were asked to respond to the statement “my hypothyroidism medication controls my symptoms well”, with the following response options: "strongly disagree", "tend to disagree", “neither agree nor disagree”, “tend to agree”, “strongly agree”, and “uncertain”)

Confidence and trust in healthcare staff (participants were asked to respond to the question: “do you have confidence and trust in the healthcare staff treating your hypothyroidism?”, with the following response options: “yes, always”, “yes, sometimes”, and “no”)

Satisfaction with care and treatment for hypothyroidism (participants were asked “how satisfied are you with the overall care and treatment you have received for your hypothyroidism?”, with the following response options: “very satisfied”, “slightly satisfied”, “neither satisfied nor dissatisfied”, “slightly dissatisfied”, “very dissatisfied” and “don’t know”)

Impact of hypothyroidism on daily living (participants were asked to respond to the statement “my hypothyroidism has affected everyday activities that people my age usually do (e.g. exercise, household chores, etc.)”, with the following response options: "strongly disagree", "tend to disagree”, “neither agree nor disagree”, “tend to agree”, “strongly agree”, and “uncertain”). For more information on Supplementary Material and for details on the different file types accepted, please see [here](https://www.frontiersin.org/guidelines/author-guidelines#supplementary-material).

**SUPPLEMENT 3**

Number of questions answered correctly, for respondents answering all questions. The vertical axis showed percentage of correct respondents. The horizontal axis shows the number of statements that were answered correctly.

## Supplementary Figures

**
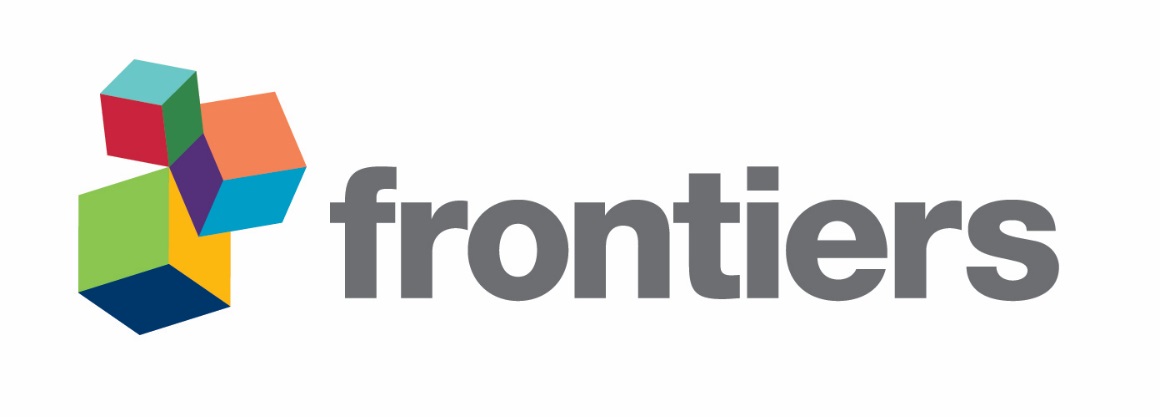
**

**Supplementary Figure 1.** The figure legends are required to have the same font as the main text, 12 point normal Times New Roman, single spaced. Please use a single paragraph for each legend and prepare the figures keeping in mind the PDF layout.
