# Supplementary material for: Knowledge gaps about the diagnosis and treatment of hypothyroidism: an international patient survey
Source: Front Endocrinol (Lausanne). 2025 Aug 29;16:1663497. doi: 10.3389/fendo.2025.1663497 (PMC12425718; doi:10.3389/fendo.2025.1663497)
Supplement: Supplementary file 2 [file DataSheet2.docx]

Supplementary Material

# Supplementary Data

**SUPPLEMENT 2**

Independent variables used in the study. Where necessary, we recoded the survey data into a smaller number of categories to meet the assumptions of the chi square tests; for instance classifying “well above average” and “above average” to “above average” for the household income variable. With regards to anxiety and low mood / depression, participants were asked “during the past 4 weeks, how much have you been bothered by anxiety?” and “during the past 4 weeks, how much have you been bothered by low mood / depression?” with the following response options: “bothered a little” or “bothered a lot” (considered as having anxiety or low mood / depression), “not bothered at all” (considered as not having anxiety or low mood / depression).

Demographics

Gender

Age

Marital status

Employment status

Ethnic background

Countries

Years in education

Household income

Clinical variables

Number of comorbidities

Duration of hypothyroidism

Cause of hypothyroidism

Most recent serum TSH concentration

Highest ever recorded serum TSH

Treatment for hypothyroidism

Probable Somatic Symptom Disorder

Type D personality

Anxiety

Low mood/depression

Use of internet and social media to find information about hypothyroidism

Patient reported outcomes

Symptom control by treatment for hypothyroidism; participants were asked to respond to the statement “my hypothyroidism medication controls my symptoms well”, with the following response options: "strongly disagree", "tend to disagree", “neither agree nor disagree”, “tend to agree”, “strongly agree”, and “uncertain”)

Confidence and trust in healthcare staff (participants were asked to respond to the question: “do you have confidence and trust in the healthcare staff treating your hypothyroidism?”, with the following response options: “yes, always”, “yes, sometimes”, and “no”)

Satisfaction with care and treatment for hypothyroidism (participants were asked “how satisfied are you with the overall care and treatment you have received for your hypothyroidism?”, with the following response options: “very satisfied”, “slightly satisfied”, “neither satisfied nor dissatisfied”, “slightly dissatisfied”, “very dissatisfied” and “don’t know”)

Impact of hypothyroidism on daily living (participants were asked to respond to the statement “my hypothyroidism has affected everyday activities that people my age usually do (e.g. exercise, household chores, etc.)”, with the following response options: "strongly disagree", "tend to disagree”, “neither agree nor disagree”, “tend to agree”, “strongly agree”, and “uncertain”).
