# Supplementary material for: Knowledge gaps about the diagnosis and treatment of hypothyroidism: an international patient survey
Source: Front Endocrinol (Lausanne). 2025 Aug 29;16:1663497. doi: 10.3389/fendo.2025.1663497 (PMC12425718; doi:10.3389/fendo.2025.1663497)
Supplement: Supplementary file 3 [file DataSheet3.docx]

Supplementary Material

# Supplementary Data

**SUPPLEMENT 3**

Number of questions answered correctly, for respondents answering all questions. The vertical axis showed percentage of correct respondents. The horizontal axis shows the number of statements that were answered correctly.
