# Supplementary material for: Knowledge gaps about the diagnosis and treatment of hypothyroidism: an international patient survey
Source: Front Endocrinol (Lausanne). 2025 Aug 29;16:1663497. doi: 10.3389/fendo.2025.1663497 (PMC12425718; doi:10.3389/fendo.2025.1663497)
Supplement: Supplementary file 4 [file DataSheet4.docx]

Supplementary Material

# Supplementary Data

**SUPPLEMENT 4**

Associations of “Incorrect” responses to principal knowledge statement (“a patient with a normal thyroid blood test does not need to be treated with thyroid hormones (even if they have positive thyroid antibodies and symptoms ) with variables. The Bonferroni adjusted threshold was 0.0022.

|  | **Chi-squared statistic** | **P-value** | **Adjusted Significance** |
| --- | --- | --- | --- |
| Gender | 4.449 | 0.349 | Not Significant |
| Country | 101.823 | 5.93E-13 | Significant |
| Age | 11.632 | 0.310 | Not Significant |
| Marital status | 2.793 | 0.593 | Not Significant |
| Ethnicity | 5.664 | 0.932 | Not Significant |
| Years in education | 12.624 | 0.049 | Not Significant |
| Household income | 16.167 | 0.040 | Not Significant |
| Employment status | 12.578 | 0.050 | Not Significant |
| Comorbidities | 11.341 | 0.023 | Not Significant |
| Duration of hypothyroidism | 4.230 | 0.376 | Not Significant |
| Cause of hypothyroidism | 67.741 | 1.21E-10 | Significant |
| Most recent serum TSH | 80.073 | 4.73E-14 | Significant |
| Highest ever recorded serum TSH | 7.245 | 0.123 | Not Significant |
| Treatment type for hypothyroidism | 189.891 | 2.68E-38 | Significant |
| Probable Somatic Symptom Disorder | 9.221 | 0.010 | Not Significant |
| Type D personality | 3.817 | 0.701 | Not Significant |
| Anxiety | 6.128 | 0.047 | Not Significant |
| Low mood /depression | 11.746 | 0.003 | Not Significant |
| Use of internet and social media | 119.321 | 4.58E-22 | Significant |
| Hypothyroidism symptom control with medication | 15.689 | 3.92E-04 | Significant |
| Confidence and trust in healthcare staff | 40.572 | 1.55E-09 | Significant |
| Satisfaction with overall care and treatment for hypothyroidism | 30.915 | 1.94E-07 | Significant |
| Impact of hypothyroidism on everyday activities | 26.769 | 1.54E-06 | Significant |
