# Supplementary material for: Knowledge gaps about the diagnosis and treatment of hypothyroidism: an international patient survey
Source: Front Endocrinol (Lausanne). 2025 Aug 29;16:1663497. doi: 10.3389/fendo.2025.1663497 (PMC12425718; doi:10.3389/fendo.2025.1663497)
Supplement: Supplementary file 5 [file DataSheet5.docx]

Supplementary Material

# Supplementary Data

**SUPPLEMENT 5**

Heatmap for significant associations between “Incorrect” responses to principal knowledge statement and other variables, based on the differences between observed and expected frequencies (figure for difference between observed and expected are shown in the columns). The red colour represents frequencies between variables that were fewer than expected and blue more than expected. The intensity of color is proportional to the size of the difference of observed data from expectation. “Incorrect” group: incorrect response, “Correct” group: correct response, “Unsure” group: selected “don’t know”. Abbreviations: L-T4: levothyroxine; L-T3, liothyronine, DTE: desiccated thyroid extract.
