# Supplementary material for: Knowledge gaps about the diagnosis and treatment of hypothyroidism: an international patient survey
Source: Front Endocrinol (Lausanne). 2025 Aug 29;16:1663497. doi: 10.3389/fendo.2025.1663497 (PMC12425718; doi:10.3389/fendo.2025.1663497)
Supplement: Supplementary file 6 [file DataSheet6.docx]

Supplementary Material

# Supplementary Data

**SUPPLEMENT 6**

Example trees from the Gradient Boosted Decision Tree model. These individual trees are considered “weak learners” and are used in silico as an ensemble to make the final predictions. However, that model is only moderately performative (AUC = 0.65)
