# Supplementary material for: Knowledge gaps about the diagnosis and treatment of hypothyroidism: an international patient survey
Source: Front Endocrinol (Lausanne). 2025 Aug 29;16:1663497. doi: 10.3389/fendo.2025.1663497 (PMC12425718; doi:10.3389/fendo.2025.1663497)
Supplement: Supplementary file 7 [file DataSheet7.docx]

Supplementary Material

# Supplementary Data

**SUPPLEMENT 7**

Predictive performance for Gradient Boosted Decision Tree GBDT method, applied to the test data sub-sample. Responses to the principal knowledge statement in groups: “Incorrect”, “Correct” and “Unsure”.

|  | **Precision** | **Recall** | **F1-score** |
| --- | --- | --- | --- |
| **Metrics for “Incorrect”** | 0.574 | 0.817 | 0.674 |
| **Metrics for “Correct”** | 0.231 | 0.016 | 0.030 |
| **Metrics for “Unsure”** | 0.511 | 0.413 | 0.457 |
