# Supplementary material for: Knowledge gaps about the diagnosis and treatment of hypothyroidism: an international patient survey
Source: Front Endocrinol (Lausanne). 2025 Aug 29;16:1663497. doi: 10.3389/fendo.2025.1663497 (PMC12425718; doi:10.3389/fendo.2025.1663497)
Supplement: Supplementary file 8 [file DataSheet8.docx]

Supplementary Material

# Supplementary Data

**SUPPLEMENT 8**

The table shows data for respondents that provided incorrect responses (“Incorrect” group) in relation to the principal knowledge statement. The proportion of such respondents who also provided incorrect (“Incorrect” group), correct (“Correct group) answers or were unsure (“Unsure group) to the remaining statements is shown in the right-hand columns. The denominators in calculating percentages excluded missing data.

| **Knowledge statements** | **“Incorrect” group**  **% (n)** | **“Correct” group**  **% (n)** | **“Unsure” group**  **% (n)** |
| --- | --- | --- | --- |
| It's safe to be slightly over-treated with thyroid hormones (e.g. having a TSH below the normal range) | 46.2 (818/1769) | 34.3 (606/1769) | 19.5 (345/1769) |
| Body temperature is the best method for diagnosing hypothyroidism | 10.0 (177/1769) | 58.8 (1041/1769) | 31.1 (551/1769) |
| Most patients with untreated hypothyroidism also have problems with their adrenal glands | 29.9 (528/1766) | 9.7 (171/1766) | 60.4 (1067/1766) |
| You can manage your hypothyroidism without medication, just by watching what you eat | 5.4 (94/1751) | 82.1 (1437/1751) | 12.6 (220/1751) |
| Hypothyroidism is an infectious disease | 1.9 (34/1751) | 94.7 (1658/1751) | 3.4 (59/1751) |
| Hypothyroid patients can lose weight if adequately treated | 20.3 (355/1751) | 63.2 (1106/1751) | 16.7 (290/1751) |
| Hypothyroid patients need to take iodine supplements even if they are already taking thyroid hormone | 7.6 (134/1753) | 62.6 (1098/1753) | 29.7 (521/1753) |
